# N-acetyl cysteine induces quiescent-like pancreatic stellate cells from an active state and attenuates cancer-stroma interactions

**DOI:** 10.1186/s13046-021-01939-1

**Published:** 2021-04-15

**Authors:** Haimin Feng, Taiki Moriyama, Kenoki Ohuchida, Nan Sheng, Chika Iwamoto, Koji Shindo, Kengo Shirahane, Naoki Ikenaga, Shuntaro Nagai, Kohei Nakata, Kazuhiro Mizumoto, Masafumi Nakamura

**Affiliations:** 1 Department of Surgery and Oncology, Graduate School of Medical Sciences, Kyushu University, 3-1-1 Maidashi, Higashi-ku, Fukuoka, 812-8582 Japan; 2 Department of Endoscopic Diagnostics and Therapeutics, Kyushu University, 3-1-1 Maidashi, Higashi-ku, Fukuoka, 812-8582 Japan; 3 Cancer Center of Kyushu University Hospital, 3-1-1 Maidashi, Higashi-ku, Fukuoka, 812-8582 Japan

**Keywords:** N-acetyl-cysteine, Pancreatic Cancer, Pancreatic stellate cells, Pioglitazone, Cancer-stromal interactions

## Abstract

**Background:**

Pancreatic stellate cells (PSCs) occupy the majority of the pancreatic cancer microenvironment, contributing to aggressive behavior of pancreatic cancer cells (PCCs). Recently, anti-fibrotic agents have proven to be an effective strategy against cancer, but clinical trials have shown little efficacy, and the driving mechanism remains unknown. N-acetyl-cysteine (NAC) is often used for pulmonary cystic fibrosis. Pioglitazone, an agonist of peroxisome proliferator-activated receptor gamma, was habitually used for type II diabetes, but recently reported to inhibit metastasis of PCCs. However, few studies have focused on the effects of these two agents on cancer-stromal interactions.

**Method:**

We evaluated the expression of α-smooth muscle actin (α-SMA) and the number of lipid droplets in PSCs cultured with or without NAC. We also evaluated changes in invasiveness, viability, and oxidative level in PSCs and PCCs after NAC treatment. Using an indirect co-culture system, we investigated changes in viability, invasiveness, and migration of PSCs and PCCs. Combined treatment effects of NAC and Pioglitazone were evaluated in PSCs and PCCs. In vivo, we co-transplanted KPC-derived organoids and PSCs to evaluate the effects of NAC and Pioglitazone’s combination therapy on subcutaneous tumor formation and splenic xenografted mouse models.

**Results:**

In vitro, NAC inhibited the viability, invasiveness, and migration of PSCs at a low concentration, but not those of PCCs. NAC treatment significantly reduced oxidative stress level and expression of α-SMA, collagen type I in PSCs, which apparently present a quiescent-like state with a high number of lipid droplets. Co-cultured PSCs and PCCs mutually promoted the viability, invasiveness, and migration of each other. However, these promotion effects were attenuated by NAC treatment. Pioglitazone maintained the NAC-induced quiescent-like state of PSCs, which were reactivated by PCC-supernatant, and enhanced chemosensitivity of PCCs. In vivo, NAC and Pioglitazone’s combination suppressed tumor growth and liver metastasis with fewer stromal components and oxidative stress level.

**Conclusion:**

NAC suppressed activated PSCs and attenuated cancer-stromal interactions. NAC induces quiescent-like PSCs that were maintained in this state by pioglitazone treatment.

**Supplementary Information:**

The online version contains supplementary material available at 10.1186/s13046-021-01939-1.

## Introducion

Pancreatic ductal adenocarcinoma (PDAC), which is the ninth most common cancer, causes fourth common in cancer-related deaths in America [1]. Although great strides have been made to explore the mechanism of pathogenesis in the past decade, the 5-year survival rate of PDAC is still less than 7% because of the high rate of resistance to chemotherapy and metastasis [2], which indicates that a better treatment strategy should be developed to improve the prognosis of PDAC.

There is a growing consensus has been accepted that the tumor microenvironment may be a significant contributing factor to the aggressive behavior of cancer and its resistance to chemotherapy [3]. PDAC is notable for its excessive desmoplasia that is caused predominately by pancreatic stellate cells (PSCs) [4], which are usually in a quiescent state containing a large amount of vitamin A stored in lipid vacuoles [5]. They are activated and become myofibroblast-like cells by various growth factors secreted from pancreatic cancer cells (PCCs), which include platelet-derived growth factor (PDGF) and vascular endothelial growth factor (VEGF) [6]. As a result, activated PSCs express α-smooth muscle actin (α-SMA) and secrete several cytokines and chemokines, such as IL-6, PDGF, TGF-β, and connective tissue growth factor (CTGF), which promote the invasion ability of PCCs [7]. Activated PSCs also secrete an abundant extracellular matrix (ECM), inducing immunosuppression and chemoresistance [3, 8]. Thus, targeting cancer-stroma interactions (PCCs-PSCs) appears to be a promising therapeutic strategy. It has been recently reported that inhibiting Hedgehog signals in tumor stromal cells retards the growth and metastasis of PCCs [9]. However, some research revealed that targeting tumor stroma can also promote cancer cell aggressiveness [10, 11]. Therefore, owing to the controversial nature of stromal-targeting therapeutic approaches, PSCs’ precise characteristics are still under investigation.

N-acetyl-cysteine (NAC) is an aminothiol and synthetic precursor of de novo Glutathione (GSH) synthesis [12]. NAC is also commonly applied to ameliorate inflammation under pathological conditions such as chronic obstructive pulmonary disease, influenza, and idiopathic pulmonary fibrosis [13, 14]. NAC also inhibits the activation of transcription factor activities like JNK, p38 MAPK, and NF-κB that regulate numerous genes’ expression [15]. Besides, several reports indicate that NAC induces apoptosis in colon carcinoma cells [16]. We have previously shown that some anti-fibrosis agents, such as Pirfenidone and calpeptin, have the ability to suppress pancreatic cancer by disrupting cancer-stromal interactions [17, 18]. Enhanced desmoplastic responses by coadministration of Pirfenidone and NAC have been observed in HapT1-derived orthotopic tumors in a hamster model [19]. However, few studies explored the effects of NAC on cancer-stromal interactions or the functional alterations of PSCs in PDAC.

In this study, we hypothesized that NAC might be an effective anti-fibrosis against pancreatic cancer and alter the function of PSCs. The present data revealed that low concentrations of NAC suppressed activation of PSCs and reduced its oxidative stress level. NAC-treated PSCs had a quiescent-like state that was maintained by coadministration of NAC and Pioglitazone, a ligand of peroxisome proliferator-activated receptor gamma (PPARγ) which used for type II diabetes previously [20]. Activated PSCs significantly increased the cell viability and invasiveness abilities of PCCs. Additionally, PCCs also promoted the cell viability, migration ability and oxidative stress levels of PSCs, but these mutual promotion effects were effectively attenuated by NAC treatment. The effects of combined of NAC and PLZ were showed superiority of treatment than Gemcitabine on subcutaneously transplanted mouse model, which were co-implanted with PDAC drived organoids and PSCs. Moreover, we used a splenic xenografted model to evaluate the effects of this combined treatment. Combined treatment with NAC and Pioglitazone may be a novel therapy that targets cancer-stromal interactions.

## Materials and methods

### Cell and reagents

Human primary activated PSCs were harvested from surgical specimens of pancreatic cancer, established and maintained as described by Bachem et al. [21–23] . The identity of the isolated PSCs was confirmed by their fibroblast-like morphology, positive staining for α-SMA and negative for cytokeratin 19 (CK19) (Fig. S1), though there are a tiny fraction of the fibroblast-like cells express very low levels of α-SMA. Three primary cultures of PSCs were used within eight passages in this study. Three human PCC lines were also used in this study: PANC-1 (Riken Bioresource Center, Ibaraki, Japan), SUIT-2, and BxPC-3 (National Kyushu Cancer Center, Fukuoka, Japan). All cells were maintained in Dulbecco’s modified Eagle’s medium (DMEM) supplemented with 10% fetal bovine serum and maintained at 37 °C with 10% CO_2_.

Agents included NAC, chloroquine (CQ), JQ-1, Pioglitazone (PLZ), Cryptotanshinone (CTS), and Butylated hydroxyanisole (BHA) were purchased from Sigma-Aldrich (Tokyo, Japan). We also used Anti-IL-6 neutralizing antibody (69001–1-IG, Proteintech Group, Inc.) and human recombination IL-6 proteins (AF-200-06, PeproTech) in this study. For in vitro experiments, NAC was dissolved in PBS at 100 mM and other agents were dissolved in DMSO at 1 mM. All stock solutions were stored at − 20 °C until use. Additionally, 400 μL of 100 mM NAC-containing PBS was added to 16 mL DMEM for a final concentration of 2.5 mM (pH =7.55). There was no noticeable change in pH after adding NAC to the culture medium, which confirmed that the buffering was sufficient to maintain a stable pH.

### PDAC organoid culture

To establish PDAC organoids, we harvested and digested surgical specimens of PDAC patients with a Tumor Dissociation Kit (human, Cat#130–095-929; Miltenyi Biotec, CA, USA) and cultured them using the method as described previously [24]. Briefly, the digested tumor tissue was integrated into a growth factor-reduced Matrigel (Cat# 356231; BD Bioscience, CA, USA), and finally cultured in a human complete medium 37C° for 2 weeks.

### Production of conditioned media (CM)

To exclude the effects of growth factors in FBS, we prepared conditioned media using supernatants (SNs) from PSCs and PCCs cultured in serum-free DMEM. SNs were passed through an Amicon Ultra-15 30 K Centrifugal Filter Device (Millipore, Billerica, MA, USA) in accordance with the manufacturer’s protocol. In brief, subconfluent (75% confluence) SUIT-2 cells, PSCs, or 2.5 mM NAC-treated PSCs were cultured in serum-free DMEM for 48 h. Then, 12 mL of culture supernatant was collected in the filter device, centrifuged at 5000 g in a fixed-angle rotor for 30 min followed by recovery with 10 mL of 11.2 mM NaCl. The conditioned media [CM; also defined as supernatant (SN)] was collected after removal of salts and stored at − 20 °C until use. SN from SUIT-2 (SUIT2-SN) or NAC-treated SUIT-2 (NAC-SUIT2-SN) were used to determine the effects on PSCs in cell viability and migration assays. SN from PSCs (PSC-SN) or NAC-treated PSCs (NAC-PSC-SN) was used in cell viability assays, migration assays, and gemcitabine resistance experiments. DMEM was centrifuged and concentrated as a control.

### Immunofluorescence staining

Cells were plated in glass-bottomed dishes (ibidi, Munich, Germany) at 1 × 10^5^ cells/well and treated with NAC or Control (PBS) for 48 h, then fixed with − 20 °C ethanol, blocked with 3% BSA in PBS and incubated with 10 mg/mL indicated primary antibodies at 4 °C overnight. These primary antibodies were used: anti-α-SMA (1:100), anti-CK19 (1:100), or anti-vimentin (1:100). The corresponding 10 mg/mL secondary antibodies carrying green- and red-fluorescent dye and 1 mg/mL nuclear DNA binding 4′,6-diamidino-2-phenylindole (DAPI; Dojindo, Kumamoto, Japan) were applied for protein marking. They were incubated at room temperature for 60 min and then washed with 0.1% BSA, detected by a fluorescence microscope (BZ-X710; Keyence).

### Cell viability assays

We performed a CellTiter-Glo viability assay (Promega; Madison, WI) to measure the viability of PSCs and PCCs in accordance with the manufacturer’s protocol. Cells were seeded in 96-well plates (Greiner Bio-One, Kremsünster, Austria) in triplicate at 1 × 10^3^ cells/well in 100 μL fresh DMEM containing 10% FBS. The background was subtracted using values from wells containing only culture medium. To assess stimulation effects, the medium was replaced with medium containing NAC, PBS, or each SN as indicated in the figure legends at 24 h after seeding. For all cell viability assays, data are representative of three independent biological experiments.

### Invasion and migration assays

The ability of migration and invasion in PSCs and PCCs were performed by counting the number of cells migrated or invaded through a Transwell System (8-mm pore size; Becton Dickinson, Franklin Lakes, NJ). For monoculture or co-culture migration and invasion assays, we seeded cells and treated them as previously described [25]. For a collagen-coated invasion assay, membranes were coated with 100 μl collagen type I (354,236; Corning, America), type IV collagen (354,233; Corning), or Matrigel at 20 mg per well. To assess the effects of PSC and PCC supernatant stimulation on migration and invasion, separate batches of cells were cultured with each SN indicated in the figure legends. At 24 h (for migration) and 48 h (for invasion) after cell seeding, migrated or invaded cells were fixed with 70% ethanol, stained with hematoxylin and eosin, and counted in five random fields (× 100 magnification). In some cases, migration and invasion abilities were evaluated by normalization to total cell numbers of each cell type to avoid the effects of proliferated cell numbers on migration and invasion. For all migration and invasion assays, three experiments were independently conducted in triplicate.

### Assessment of oxidative stress response

The levels of total glutathione (GSH) and intracellular ROS (Reactive oxygen species) were measured using an Oxiselect Total Glutathione Assay Kit (STA-312, Cell Biolabs Inc., San Diego, CA, USA) and an Oxiselect intracellular ROS Assay Kit (STA-342, Cell Biolabs) according to the manufacturer’s instructions. The total glutathione concentration in the samples was measured by recording the absorbance at 405 nm with a microplate reader and analyzed by comparison to a standard curve. Intracellular ROS levels were measured in terms of relative fluorescence units (RFUs) at excitation and emission wavelengths of 485 nm and 535 nm. All experiments were independently performed three times.

### Lipid droplet accumulation assay

Lipid droplet accumulation in PSCs was analyzed by staining with bodipy (#D-3922; Life Technologies) as described previously [26]. After staining, images were captured in three random fields using a fluorescence microscope, and we counted the number of bodipy-positive punctures per cell in 20 cells.

### qRT-PCR analysis

Total RNA of cells was extracted using a High Pure RNA Isolation kit (Roche, Mannheim, Germany). We performed qRT-PCR using a QuantiTect SYBR Green RT-PCR kit (Qiagen, Tokyo, Japan) and the CFX96 Real-Time PCR System (Bio-Rad Laboratories, Hercules, CA). 18S rRNA was used to normalize mRNA expression. All primers were purchased from Sigma-Aldrich. For details, see Supplementary Table S1. Three experiments were independently conducted in triplicate.

### Western blotting analysis

Total cellular proteins of PSCs and PCCs were prepared with PRO-PREP Protein Extraction Solution (InTron Biotechnology, Seongnam, Korea). Supernatant proteins were collected as described previously [26]. Protein samples of cell lysates (20 μg) or supernatants (30 μg) were separated by 10% SDS PAGE on Mini-PROTEAN TGX Precast Gels (Bio-Rad Laboratories), followed by transfer to Trans-Blot Turbo Mini PVDF Transfer Packs (Bio-Rad Laboratories). The membrane was incubated at 4 °C overnight with primary antibodies (1:1000; supplementary Table S2) and then probed with secondary antibodies (1:2000; supplementary Table S2). Immunoblots were developed by ECL western blotting detection reagent (Bio-Rad) and analyzed with the ChemiDoc XRS System (Bio-Rad Laboratories).

### Small interfering RNA silencing of PPARγ

PSCs or NAC-PSCs at 90% confluence were transfected with siPPARγ-1(#SI02634275, GS5468, Qiagen, Hilden, Germany) and siPPARγ-2 (#SI00071680) small interfering RNA (siRNA) by electroporation using a Nucleofector System (Lonza, Basel, Switzerland) according to the manufacturer’s recommendations. To verify knockdown specificity, we used a control siRNA (#1027310). Transfected cells were used in subsequent experiments 24–72 h after transfection. Three separate PSC preparations were used.

### In vivo experiments

For in vivo experiments, we used 4-week-old female nude mice (Kyudo Co., Saga, Japan) under the approval of the Ethics Committee of Kyushu University. For subcutaneous transplantation experiments, PDAC organoids (5 × 10^5^) with PSCs (5 × 10^5^) were suspended in 100 μL DMEM and subcutaneously transplanted into the left flank of mice. One week after implantation (day 7), mice were intraperitoneally injected with Control (100 μl PBS + 0.1 μl DMSO, twice a week), GEM (40 mg/kg, once a week) or NAC (500 mg/kg) with PLZ (4 mg/kg) twice a week for 4 weeks and then sacrificed on day 35. For splenetic metastasis experiments, nude mice underwent splenic implantation of PDAC organoids (1 × 10^5^) with PSCs (1 × 10^5^). One week after implantation (day 7), mice were injected intraperitoneally with Control (100 μl PBS + 0.1 μl DMSO), NAC (500 mg/kg), PLZ (4 mg/kg), or co-treatment (NAC + PLZ) once every 2 days for 3 weeks and sacrificed on day 28. All orthotopic tumors and liver tissues were resected and weighed. We estimated tumor volume by the following formula: π/6 × (L × W × W) (L = the largest tumor diameter, W = the smallest tumor diameter) [17]. Mouse tumor tissues were prepared to 4-μm-thick paraffin sections for experimental validation at the tissue levels.

### Immunohistochemistry

For immunohistochemistry (IHC) staining, the following primary antibodies were used: α-SMA (1:500), CK 19 (1:200), PCNA (1:500), PPARγ (1:100), HMOX-1 (1:200) and Nrf2 (1:100). Tissues were immunostained as described previously [27]. Masson’s trichrome staining was performed by a ready-to-use kit (Sigma-Aldrich). To calculate the positive index of each staining, three random fields were counted by microscope (BZ-X710; Keyence). Serial sections were used for immunohistochemical staining.

### Statistical analysis

For in vitro experiments, results are expressed as means ± SD. Significances between two groups were assessed by one-way analysis of the variance or the Student’s t-test using GraphPad Prism 7.0 (GraphPad Software). Values of *P* < 0.05 were considered statistically significant in all analyses. Survival analyses were conducted using the Kaplan-Meier method. All experiments were performed at least three times except animal experiments.

## Results

NAC inhibits the cell viability, migration, and invasion of PSCs rather than PCCs.

**Fig. 1:**
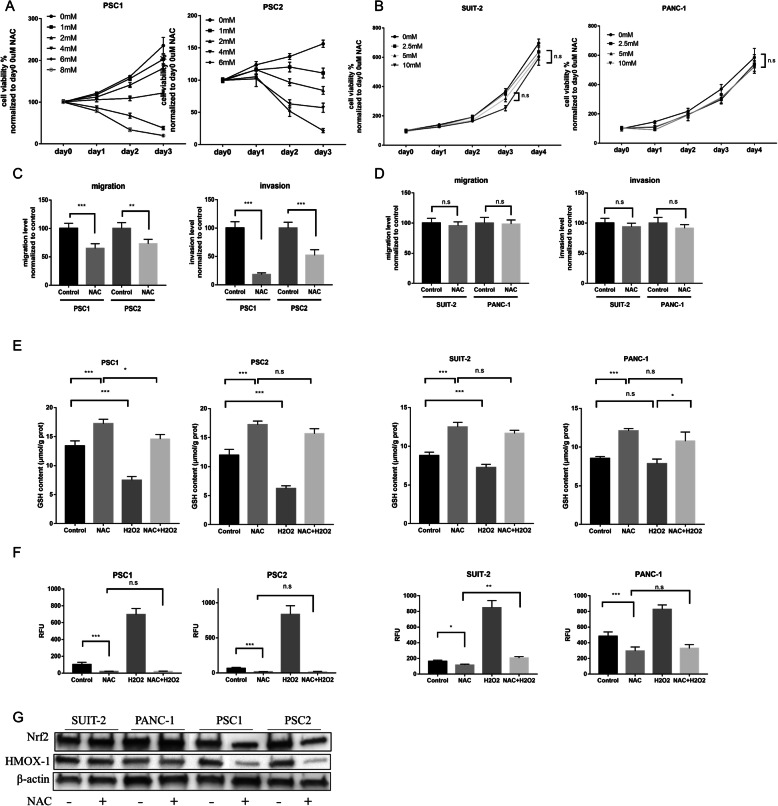
NAC inhibits the cell viability, migration, and invasion of PSCs rather than PCCs. **a**-**b** Cell viability assays of PSCs (PSC1 and PSC2) (**a**) and PCCs (SUIT-2 and PANC-1) (**b**) by various concentrations of NAC treatment were determined by a CellTiter-Glo luminescent cell viability assay. **c**–**d** Migration and invasion assays of PSCs. (**c**) and PCCs (**d**) after NAC treatment were analyzed. Migration and invasion values were normalized by the total cell numbers of each cell. For details, also see supplementary figure S2D-E. **e** Glutathione (GSH) level in PSC cells (left) which treated with 2.5 mM NAC with or without 10 μM H_2_O_2_, and in PCC cells (right) which treated with 5 mM NAC with or without 100 μM H_2_O_2_ for 24 h. Concentrations of H_2_O_2_ is different because of sensitivity to drug toxicity. **f** Intracellular reactive oxygen species (ROS) levels in PSCs and PCCs response to various treatment conditions. PSCs were treated with agents as follows: PBS (as control), 2.5 mM NAC, 20μM H_2_O_2_, or the combined 2.5 mM NAC with 20μM H_2_O_2_. PCCs were treated with components as follows: PBS (as control), 5 mM NAC, 200μM H_2_O_2_, or the combined 5 mM NAC with 200μM H_2_O_2_. At 30 min after treatment, intracellular ROS levels were measured in terms of relative fluorescence units (RFUs), the values of RFUs were normalized to the Control group in PSC1 cells. The values shown are means ± SEM from at least three independent experiments. Student’s *t*-tests (unpaired, unequal variance) were used for comparisons. **g** Expression of Nrf2 and HMOX-1 in PCCs and PSCs treated with or without NAC by western blotting.*P < 0.05; **P < 0.01; ***P < 0.001; n.s, no significance

First, we investigated the cell viability of both PSCs and PCCs by NAC treatment. In monoculture, NAC inhibited the viability of PSCs in a dose-dependent manner, and the IC_50_ of each primary culture of PSCs was calculated (Fig. 1a; supplementary Fig.S2A). However, the viability of PCCs was not inhibited by NAC at low concentrations (Fig. 1b; supplementary Fig.S2B). Similarly, 2.5 mM NAC also effectively inhibited the migration and invasion ability of PSCs, but not those of PCCs (Fig. 1c–d, supplementary Fig. 2 H-I). These results indicate that NAC, an inexpensive drug, can be used as an anti-tumor agent for pancreatic cancer by targeting PSCs.

Because NAC is commonly used as an antioxidant, we also examined the levels of total glutathione (GSH) and ROS, which are oxidative stress response markers, in PSCs and PCCs. As expected, NAC increased the level of GSH and decreased the ROS level in both PSCs and PCCs (Fig. 1e, f). However, when co-treated with H_2_O_2_, an oxidative stress inducer that causes oxidative stress in PSCs and PCCs, NAC still effectively inhibited oxidative stress in PSCs rather than PCCs. Besides, the expression of Nrf2, the master regulator of oxidative stress, as well as its downstream targets HMOX-1, NQO1and GCLC were also decreased by NAC treatment in PSCs but not in PCCs (Fig. 1g, Supplementary Fig. 2 J).

To confirm the effectiveness and safety of NAC for anti-tumor application, we explored the effects of various concentrations on PSCs. The high concentration of NAC had an apparent toxic effect on PSCs (Supplementary Fig. S2C). However, 1 mM NAC decreased the expression of α-SMA significantly. When PSCs were cocultured with supernatant from PCCs (SUIT2-SN), 1 mM NAC was not sufficient to decrease the expression of α-SMA (Supplementary Fig. S2F). Additionally, 1 mM NAC had little effect on the migration ability of PSCs with or without SUIT2-SN (Supplementary Fig. S2G). Although in flow cytometer the apoptosis rate of PSCs was increased after 48 h of 2.5 mM NAC treatment (Supplementary Fig. S2D), which might be due to the suppression of growing active cells and normal programmed cell death mechanism was activated; the expression of cleaved caspase-3 evaluated by western blotting, one of the best-known markers of apoptosis, showed no significant difference between normal PSCs and NAC-treated PSCs (Supplementary Fig. S2E). Thus, we used 2.5 mM to investigate its specific effects on PSCs in the following in vitro experiments in which the concentration inhibited cell viability and migration effectively (Fig. 1e).

### NAC decreases activation of PSCs and induces a quiescent-like state

**Fig. 2:**
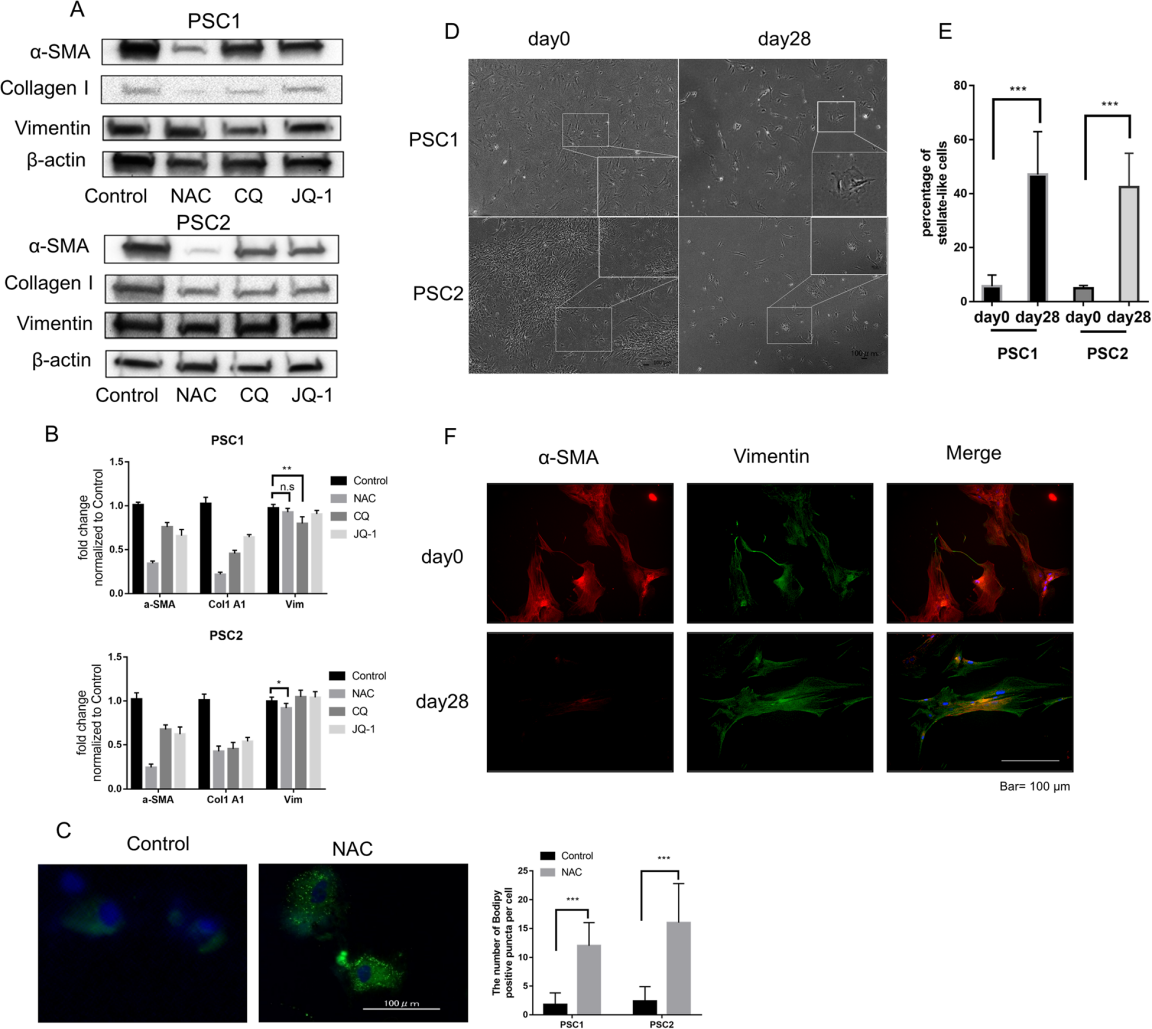
NAC decreases the activation of PSCs and induces a quiescent-like state. **a**-**b** Effect of NAC, CQ and JQ-1 treatment for 72 h were performed in PSCs on protein expression (**a**) and mRNA expression (**b**) of α-SMA, collagen type I and Vimentin. CQ and JQ-1 were used as the positive control, DMSO was used as Control (negative). **c** Lipid droplet accumulation assay of PSCs. PSCs were stained with bodipy 493/503. Quantification of bodipy-positive puncta per cell in 20 cells plotted as the mean ± SD. Scale bar =100 um. Original magnification, × 400. Also see Supplementary Fig. S3A. **d** PSCs were treated with 2.5 mM NAC in 2% FBS DMEM for 2 weeks without passaging, then cultured normally in 10%FBS DMEM for another 14 days (day28). Representative microphotographs for PSCs at days 0 (Control) and day 28 (NAC-treated) were shown. After NAC treatment, the total number of PSCs were decreased, and the number of morphologically stellate-like cells were increased. Also see Supplementary Fig.S3B (**e**) Quantitative statistics for the percentage of stellate-like cells in (**d**) were calculated in nine random fields. **f** Representative microphotograph of α-SMA (red) and vimentin (green) immunofluorescence staining in PSC1 cells at days 0 and 28. Also see Supplementary Fig. S4E for PSC2 cells. Original magnification, × 100. Scale bar = 100 μm. *P < 0.05; **P < 0.01; ***P < 0.001; n.s, no significance

Activated PSCs expressed a high level of α-SMA. We used Chloroquine (CQ) and JQ-1 as the positive control, which were reported to inhibit activation of PSCs by our previous research and Kumar K [26, 28]. Consistent with CQ and JQ-1, after NAC treatment, the expression of α-SMA and collagen type I were decreased markedly (Fig. 2a, b). However, NAC did not decrease vimentin expression, a marker commonly used to characterize the cells of fibroblast origin [29]. Moreover, treatment with NAC increased the number of lipid droplets in PSCs (Fig. 2c), which indicated a transition from activation to a quiescent-like state. These data indicate that NAC decreases the activity of PSCs.

To examine the characteristics of PSCs by NAC treatment, we established NAC-treated PSCs used in the following experiments, which were treated with NAC for 2 weeks and then cultured without NAC for another 2 weeks. After 4 weeks of culture, the number of PSCs with spindle-like morphology was decreased significantly, and the number of PSCs with star-like morphology was generally increased (Fig. 2d-e, supplementary Fig.S3B). NAC-treated PSCs (day 28) had more lipid droplets (supplementary Fig.S3A) and less expression of α-SMA compared with untreated PSCs (day 0) (Fig. 2f; supplementary Fig.S3E). Moreover, NAC-treated PSCs exhibited less cell viability and migration than untreated PSCs (Supplementary Fig. S3C, D).

### NAC attenuates cancer-stroma interaction in pancreatic cancer

To explore the effects of NAC on cancer-stroma interaction, SUIT-2 supernatant (SUIT-SN), normal PSCs supernatant (PSC-SN), and NAC-treated PSC supernatant (NAC-PSC-SN) were prepared and used for the following experiments.

**Fig. 3:**
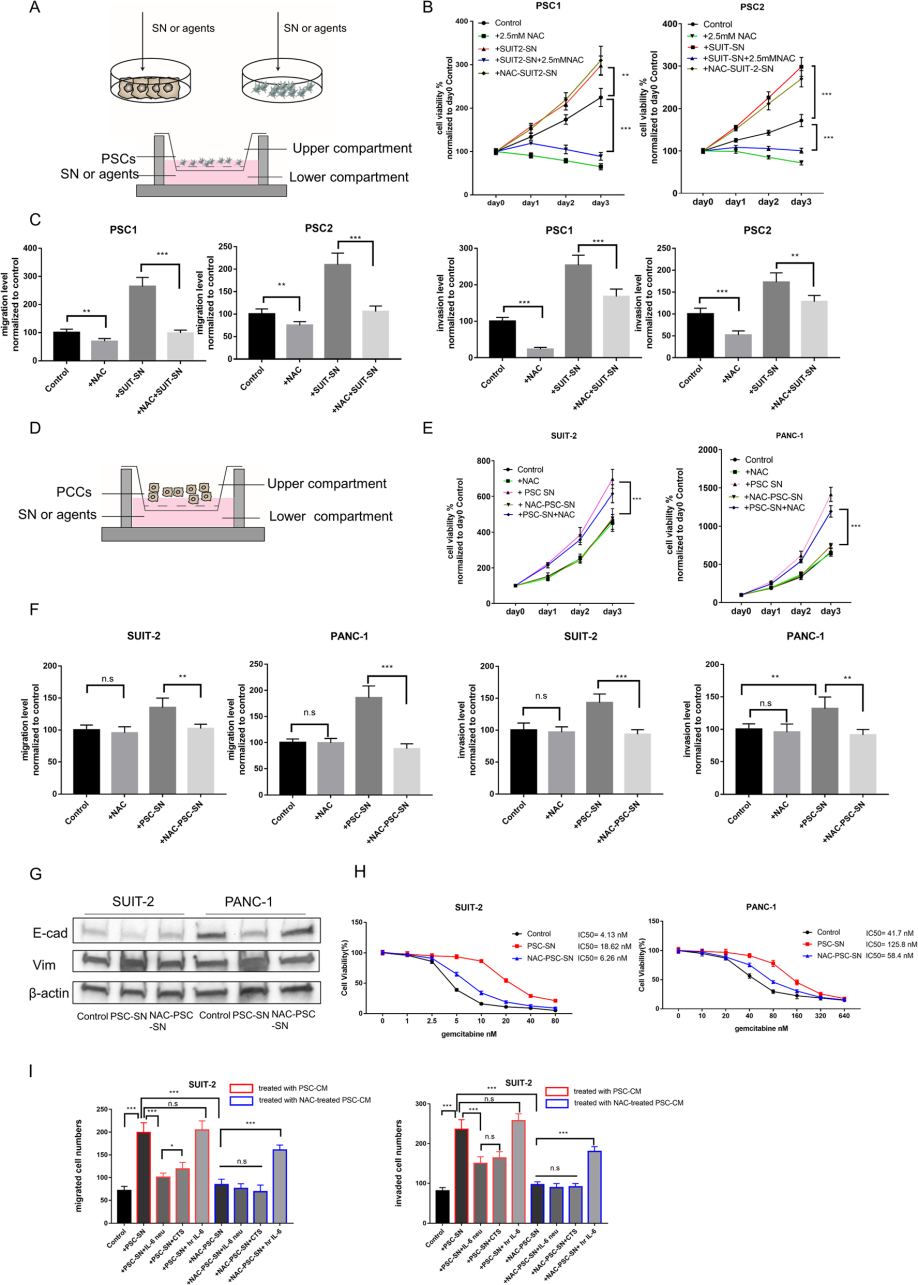
NAC attenuates cancer-stroma interaction in pancreatic cancer. **a** The schema for indirect co-culture experiments to evaluate the effects of NAC between PCCs and PSCs. (top) The supernatant (SN) was obtained as described above and then added as CM to the following experiments as indicated. (bottom) Effects of NAC in PSCs which induced by PCC-SN in the co-culture system. PCC-SN or agents were added to the plate, and PSCs were seeded in upper Chambers (8 μm). The number of migrated or invaded (**c**) PSCs were evaluated after incubation for 24 h or 48 h. **b** Cell viability assays of PSCs co-cultured with various agents. PSCs were not treated (Control) or added with 2.5 mM NAC, or SUIT2-SN, or SN from NAC-treated SUIT-2(NAC-SUIT2-SN), or dual treated with SUIT2-SN and NAC. **c** Migration and invasion assays of PSCs were performed as described in A (bottom) for 24 h or 48 h, respectively. Migrated or invaded cell numbers were normalized by the total cell number of each cell. For details, also see supplementary figure S4A. **d** The schema for effects of NAC in PCCs which induced by PSC-SN in the co-culture system. PSC-SN or agents were added to the plate, and PCCs were seeded in upper Chambers (8 μm). The number of migrated or invaded (F, I) PCCs were evaluated after incubation for 24 h or 48 h. **e** Cell viability assays of PCCs co-cultured with various agents. PCCs were not treated (Control) or added with 5 mM NAC, or PSC-SN, or SN from NAC-treated PSC (NAC-PSC-SN), or dual treated with PSC-SN and NAC. **f** Migration and invasion assays of PCCs were performed as described in D for 24 h or 48 h, respectively. Migrated or invaded cell numbers were normalized by the total cell number of each cell. For details also see supplementary figure S5. **g** Expression of E-cadherin and Vimentin in PCCS treated with PSC-SN or NAC-PSC-SN by western blotting. **h** Cell viability assays of PCCs at different concentrations of Gemcitabine after 72 h treatment. PCC cells were pre-processed with PSC-SN or NAC-PSC-SN for 48 h. Cell viability in all groups was normalized to their viability treated with 0 nM Gemcitabine. **i** Migration and invasion assays of SUIT-2 cells were performed at 24 h or 48 h after treatment with indicated agents. Migrated and invaded cells were counted. Agents were as followed: SN from PSC (PSC-SN), 5 μg/ml IL-6 neutralizing antibody (IL6 neu), 20 μM Cryptotanshinone (CTS), 20 ng/ml human recombination IL-6 (hr IL-6), SN from NAC-treated PSC (NAC-PSC-SN). Also, see supplementary figure S5C for details and figure S5D for assays of PANC-1 cells. *P < 0.05; **P < 0.01; ***P < 0.001; n.s, no significance

Firstly, we assessed the ability of cell viability, migration, and invasiveness in PSCs and PCCs after each supernatant stimulation (Fig. 3a-f; supplementary Fig.S4). SUIT-SN increased these abilities of PSCs (Fig. 3b-c; supplementary Fig.S4A); however, when PSCs were treated with SUIT-SN plus 2.5 mM NAC, these promotion effects were attenuated by NAC. Mutually, SN from PSCs have also promoted these abilities of PCCs (Fig. 3d-f; supplementary Fig.S4B), but NAC-treated PSCs did not show enhanced cell viability, migration, or invasiveness. These results were also confirmed in indirect co-culture transwell assays (supplementary Fig.S4C).

In cell viability assay, PCCs treated with PSC-SN and 5 mM NAC demonstrated no significant difference from PCCs treated with only PSC-SN (Fig. 3e). SN derived from NAC-treated PCCs still enhanced the viability of PSCs (Fig. 3b). These results indicate that NAC attenuates cancer-stroma interactions by targeting PSCs. Thus, we next examined if NAC-treated PSCs were attenuated their promotion effects on PCCs.

It has been well reported that PSCs promote migration and invasion of PCCs as well as EMT processes and resistance to chemotherapeutic agents [30]. E-cadherin expression was decreased and Vimentin was increased in PCCs cocultured with PSC-SN, but not in PCCs cocultured with NAC-PSC-SN (Fig. 3g). Additionally, PSC-SN increased the resistance of PCCs to gemcitabine. However, NAC-PSC-SN showed slightly increased resistance (Fig. 3h).

IL-6 is a central mediator of cancer cell-stromal cell interactions in PDAC. PSC-derived IL-6-activated STAT3 signaling in PCCs has been well reported in previous studies [31]. SN from PSCs, but not NAC-PSCs, increased the activation of STAT3 (Supplementary Fig. S5B). Additionally, an anti-IL-6-neutralizing antibody (IL-6 neu) and human recombination IL-6 (hr IL-6) were used to confirm activation of this pathway. To verify the effectiveness of these agents, we analyzed c-Myc (a transcriptional target of STAT3) and p21 (negatively regulated by STAT3 signaling) (Supplementary Fig. S5B). We also used a specific STAT3 inhibitor, cryptotanshinone (CTS), which significantly decreased the phosphorylation level of STAT3 in PCCs (Supplementary Fig. S5A). In PCCs, the promotive effects of PSC-SN on migration and invasion were inhibited by IL-6 neu and STAT3 inhibitor CTS. However, PSC-SN had no further promotive effects when added with human recombination IL-6 (Fig. 3i, Supplementary Fig. S5C). IL-6 neu and CTS showed no further inhibitive effects with NAC-PSC-SN, but exogenous hr. IL-6 increased the migration and invasion of PCCs cultured with NAC-PSC-SN. Thus, activated IL-6-STAT3 in cancer-stroma interactions was attenuated by NAC treatment. Taken together, these results indicate that NAC treatment has the potential to target activated PSCs and attenuate cancer-stroma interactions.

### Characteristic changes in PSCs induced by NAC treatment in cancer-stroma interaction

**Fig. 4:**
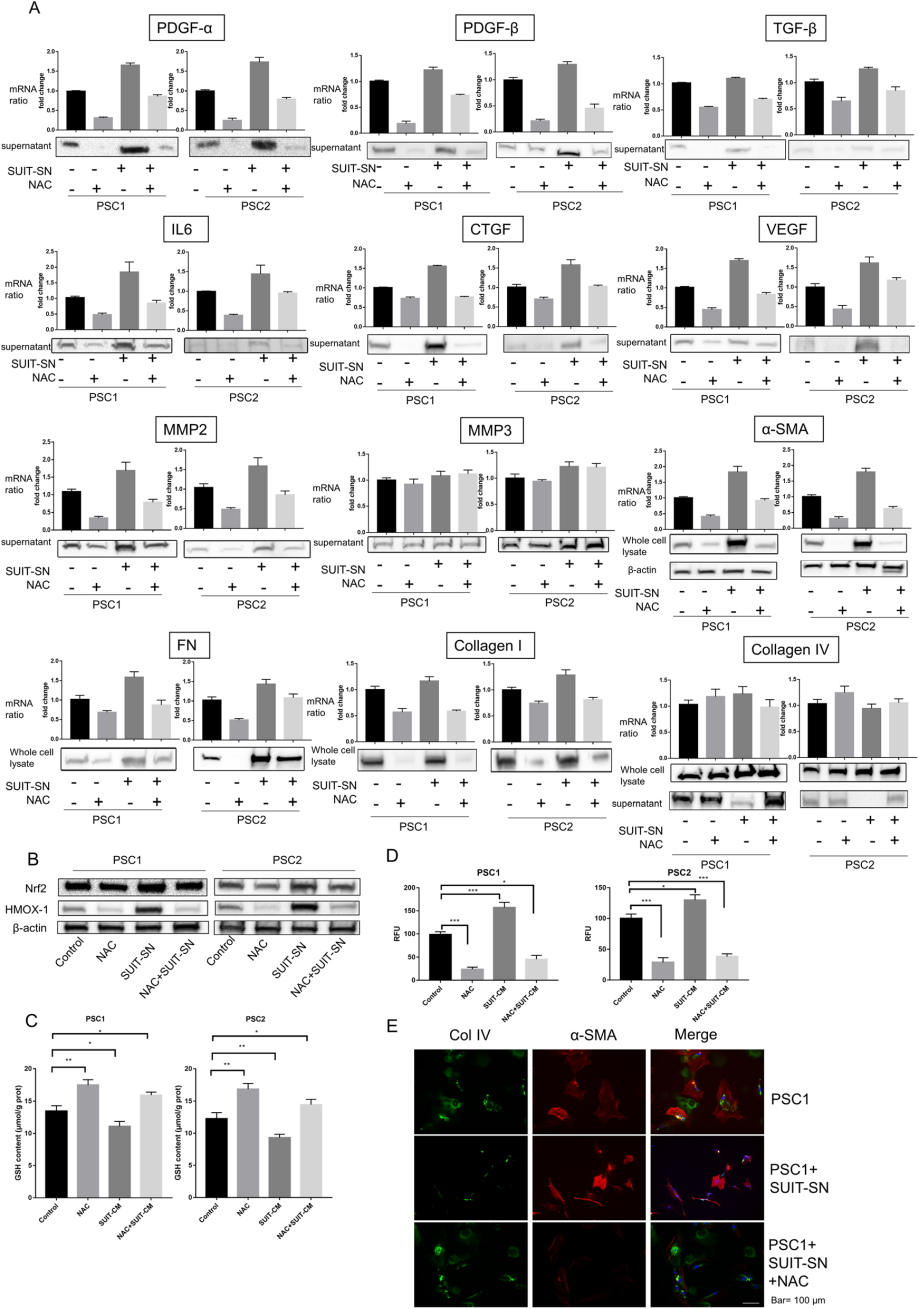
Characteristic changes in PSCs induced by NAC treatment in cancer-stroma interaction. PSCs were treated with 2.5 mM NAC, SUIT-2-SN, or SUIT-SN puls NAC, or untreated (Control). **a** The indicated expression of growth factors and ECM in PSCs were evaluated in qRT-PCR and western blotting. Protein secretion expression levels of growth factors were measured by western blotting of PSC-SN. Protein expression levels of collagen types I, collagen type IV, FN, and α-SMA was measured in whole-cell lysates with β-actin as the Control. **b** Western blotting of NRF2 and HMOX-1 (two oxidative stress response marker) levels of PSCs treated as above. **c** Glutathione (GSH) level in PSC cells treated as above. **d** Intracellular ROS levels in PSCs were treated as above. The values of RFUs (ROS level) were normalized to Control in each PSCs. **e** Representative microphotograph of α -SMA (Red) and collagen type IV (Green) immunofluorescence staining in PSC1 cells with indicated treatment. Original magnification, × 100. *P < 0.05; **P < 0.01; ***P < 0.001; n.s, no significance

To determine the secreted factors related to the inhibitory effects of NAC on cancer-stroma interactions, we evaluated mRNA and protein expression levels of those factors in PSC-SN and whole PSC cell lysates (Fig. 4a). Treatment with NAC significantly reduced the mRNA and protein levels of most secreted factors, especially PDGF-A, PDGF-B, TGF-β, CTGF, VEGF, α-SMA, FN, and collagen type I, but not collagen IV. Exposure to PCC-SN considerably increased the expression of PDGF-A, IL-6, CTGF, MMP2, α-SMA, FN, but NAC still effectively inhibited their expression. Additionally, the expression of oxidative stress response markers Nrf2 and HMOX-1 was increased by PCC-SN, but NAC inhibited their expression (Fig. 4b). In PSCs, the GSH level was increased and the ROS level was decreased by PCC-SN and NAC inhibited these changes induced by PCC-SN (Fig. 4c, d).

PCC-SN dramatically decreased collagen IV in supernatants from PSCs, although there was no significant collagen IV change in the whole-cell lysate. NAC inhibited the decrease of collagen IV in supernatants of PSCs treated with PCC-SN (Fig. 4a). These inhibitory effects were also observed by immunofluorescence staining (Fig. 4e). Compared with normal Matrix- and collagen type I-coated gels, the collagen type IV-coated gel showed a reduced number of invaded PCCs (supplementary Fig. S5E), which indicated that collagen type IV might have an anti-invasion function that was not affected by NAC. These findings suggest that NAC is an effective agent that targets tumor-stroma interactions.

**Fig. 5:**
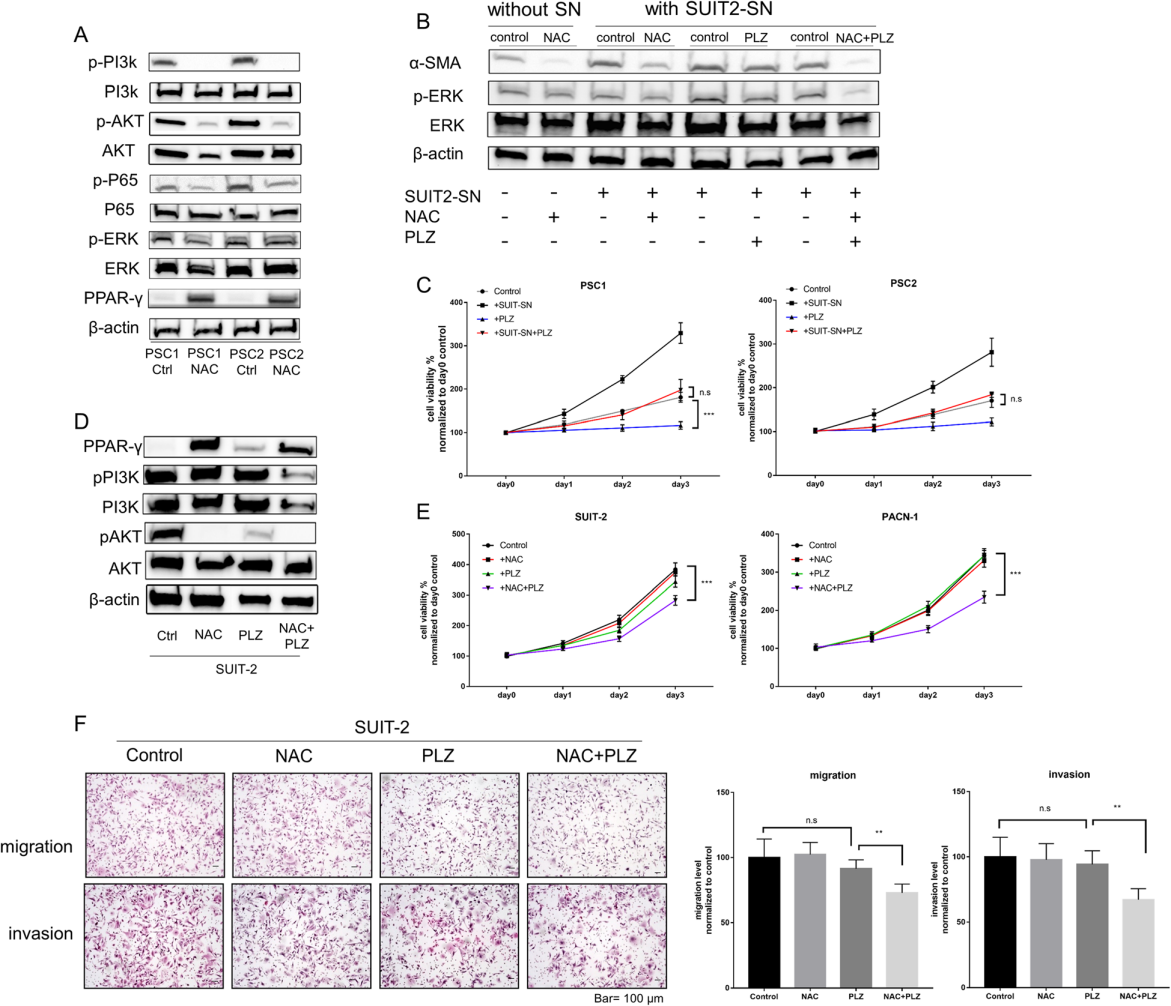
Combined NAC with PLZ suppresses reactivation of PSCs and enhances chemotherapy sensitivity of PCCs. **a** Western blotting of indicated proteins in PSC1 and PSC2 cells after treatment with NAC at the IC50 value (6.3 mM or 3.6 mM). The indicated proteins were analyzed in living adherent cells. Ctrl: control treatment with PBS. Treated PSCs were used in the following experiments. **b** Western blotting of the indicated proteins in NAC-treated PSC1 cells (indicated in lane 2) after re-treatment of indicated agents. These treatments included exposure to SUIT-SN, NAC, PLZ, or combined treatment. **c** Cell viability assays of NAC-treated PSC1 and PSC2 cells after treatment with the indicated agent. **d** Western blotting of the indicated proteins in SUIT-2 cells after the indicated treatment. NAC, 5 mM; PLZ, 20 μm; Negative Control, DMSO. **e** Cell viability assays of SUIT-2 and PANC-1 cells after treatment with indicated agents. **f** Migration and invasion assays of SUIT-2 cells treated with indicated agents (also see Supplementary figure S5E for PANC-1 cells). Migrated or invaded cell numbers were normalized by the total cell number of each cell. Original magnification, × 100. Scale bar = 100 μm. *P < 0.05; **P < 0.01; ***P < 0.001; n.s, no significance

To assess the mechanism mediated by NAC, we investigated several signaling pathways associated with the activation of PSCs. Activities of PI3K-AKT and NF-κB signaling pathways in PSCs were significantly decreased after NAC treatment. However, there was no change in ERK pathway activity (Fig. 5a). Exposure to PCC-SN increased the activities of PI3K-AKT and NF-κB signaling pathways in NAC-treated PSCs (supplementary Fig.S7A). We also found that the expression of peroxisome proliferator-activated receptor gamma (PPARγ), a nuclear receptor that regulates fatty acid storage and glucose metabolism [32], was significantly decreased (Fig. 5a). To investigate whether PPARγ plays a crucial role for inducing the in-active state in NAC-treated PSCs, we conducted small interfering RNA-mediated silencing of PPARγ in PSCs. In NAC-treated PSCs, but not untreated PSCs, the decreased expression of α-SMA was rescued by siPPARγ (Supplementary Fig. S6A). In cell viability and migration assays of PSCs, the effects were decreased in NAC-treated PSCs. However, knockdown of PPARγ attenuated these effects (Supplementary Fig. S6B, C). Because untreated PSCs expressed little PPARγ, the knockdown effects in PSCs were minimal. Furthermore, we examined the correlation between the expression of PPARγ and the oxidative stress response by cbioportal, an online tool in which RNA-seq data of 184 PDAC samples from TCGA were analyzed (Supplementary Fig. S8). High expression of PPARγ was positively associated with low expression of ACOX2, ICAM1, ICAM family, MPO, PRIMPOL, NANOS1, NOS3, and NOS1. These genes are well-reported as oxidative stress markers. These results indicated that the oxidative stress level and activation level in PSCs were decreased by NAC treatment.

### Combined NAC with PLZ suppresses reactivation of PSCs and enhances chemotherapy sensitivity of PCCs

We evaluated the therapeutic efficiency of the combination of NAC and Pioglitazone (PLZ; a ligand of PPARγ). When treated with NAC, PLZ, or both, a morphological change was observed in PSCs (supplementary Fig.S7B). PLZ (20 μM) inhibited neither the cell viability of NAC-treated PSCs directly (Fig. 5c) nor the migration and invasiveness of PSCs (supplementary Fig.S7C). NAC-treated PSCs were reactivated by SUIT-2-SN (Fig. 5b, c). However, when cotreated with 20 μM PLZ, PSCs maintained their inactive state.

The expression of PPARγ was also increased in PCCs after NAC treatment (Fig. 5d). Although low concentrations of PLZ or NAC alone did not inhibit the viability of PCCs, their co-treatment remarkably restricted the cell viability, migration, and invasiveness of PCCs (Fig. 5e, f; supplementary Fig.S7D). We also assessed downstream of PPARγ by quantitative RT-PCR. Both NAC and PLZ treatment increased the transcriptional expression of PLIN1, FABP4, Adipo, CD36, and LPL (Supplementary Fig. S8B), but not RXRα that forms a transcription factor heterodimer with PPARγ to bind to DNA elements [33]. Cotreatment with NAC and PLZ had an additive effect on these downstream targets, which promoted lipid metabolism in PSCs. To confirm whether these effects were dependent or independent of intracellular GSH levels, we used another antioxidant, butylated hydroxyanisole (BHA). BHA at 50 μM also decreased the oxidative stress level in PSCs to a similar level as 2.5 mM NAC, but its effect was less than that of NAC when cocultured with SUIT-SN (Supplementary Fig. S3F, Fig. 4d). Moreover, both BHA and NAC decreased the expression of α-SMA and increased PPARγ expression, but NAC more effectively reduced the expression of α-SMA (Supplementary Fig. S3G). These results indicate that co-treatment of NAC and PLZ may be a promising new approach for cancer treatment.

### NAC inhibits subcutaneous tumor growth in mice

To evaluate the effects of NAC on cancer-stroma interactions in vivo, we implanted SUIT-2 cells (1 × 10^6^) alone into the left flank of mice and co-implanted SUIT-2 cells with PSCs (5 × 10^5^, respectively) into the right flank of the same mice (supplementary Fig. S9A). On day 7, NAC, or PBS (Control) were injected intraperitoneally into the mice for 4 weeks. In the group without treatment, tumors in the right flank (SUIT-2 + PSC) were much larger than the left flank (SUIT-2 alone) (supplementary Fig. S9A, B). Although the left flank showed few α-SMA-positive cells, probably originated from the host, the right flank’s tumors showed much more numbers of α-SMA-positive cells and collagen fiber than the left flank (supplementary Fig. S9C, E). Treatment with NAC significantly suppressed tumor growth in both flanks compared with those of the control group (supplementary Fig. S9A, B). Compared to the control group, NAC decreased the collagen fiber areas and α-SMA positive index in both flanks (supplementary Fig. S9C-F). NAC also reduced the PCNA index in the right flank but not in the left flank than the control group (supplementary Fig. S9G). Moreover, NAC increased the PPARγ index and decreased HMOX-1 and Nrf2 index in both flanks. These findings suggest that NAC suppresses tumor formation and oxidative stress in tumor microenvironments in pancreatic cancer.

### Combined treatment of NAC with PLZ inhibits tumor growth and metastasis in KPC cancer organoid cells co-implanted with PSCs

**Fig. 6:**
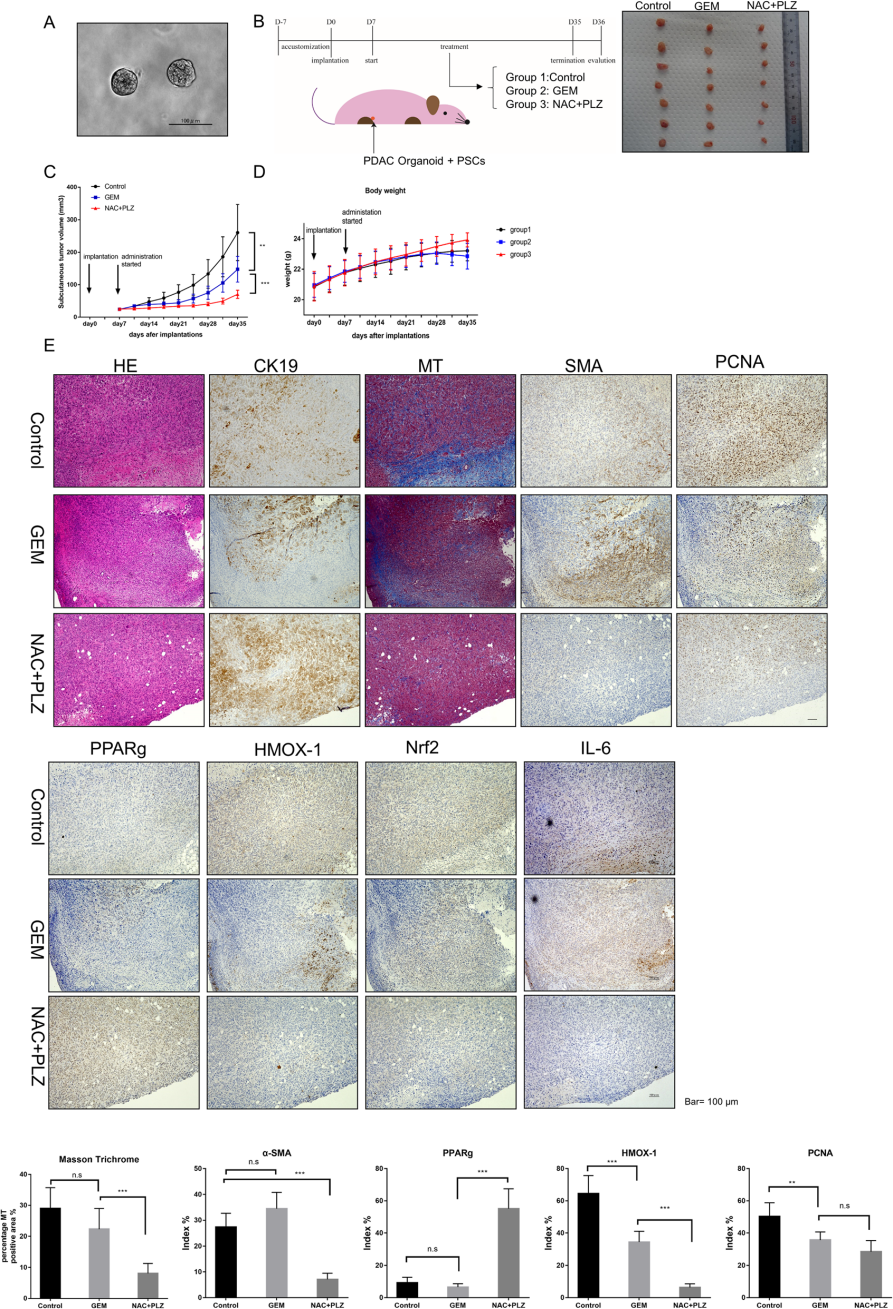
co-treatment with NAC and PLZ inhibited subcutaneous tumor growth in the xenografted organoid model with PSC co-implantation. **a** Representative photomicrographs of cancer organoids derived from KPC mice. Scale bars = 100 μm. **b** Nude mice (*n* = 7/per group) were subcutaneously transplanted with KPC mouse-derived organoids (5 × 10^5^) and mouse PSCs (5 × 10^5^) into the flank. After 1 week, the mice were injected intraperitoneally with Gemcitabine (40 mg/kg, once a week, also named group2) or NAC (500 mg/kg) + PLZ (4 mg/kg) (twice a week, group3), or Control (100 μl PBS + 0.1 μl DMSO, twice a week, group1) for 4 weeks. Representative photograph showing significantly reduced tumor formation after 4 weeks of NAC + PLZ co-treatment. **c** Tumor volume was significantly decreased in GEM and NAC + PLZ co-treatment groups after 4 weeks of treatment. **d** The average weight of each group of mice. Also see supplementary figure S6.E for weight change of each mouse in each group. **e** H&E staining, Masson’s staining trichrome and immunohistochemical staining of α-SMA, CK19, PPARγ, HMOX-1, Nrf2 and PCNA. Original magnification, × 100. Scale bar = 100 μm. *P < 0.05; **P < 0.01; ***P < 0.001; n.s, no significance

We previously established pancreatic cancer organoids for 2 weeks using PDAC cells derived from the KPC mouse (Fig. 6a). Compared with normal 2D-cultured cells, the implanted tumor showed more collagen fiber areas and more α-SMA-positive index in splenic xenograft models with 3D-organoids (supplementary Fig.S10). Thus, using this xenograft model was primely reflect tumor microenvironment in PDAC. We subcutaneously implanted KPC organoids with PSCs into nude mice treated with the control, gemcitabine (a first-line therapy for PDAC), and NAC with PLZ (Fig. 6b). After 4 weeks of treatment, the tumor volume in the cotreatment group was significantly decreased compared with GEM and control groups (Fig. 6c, Supplementary Fig. S11A). Moreover, α-SMA-positive cells and collagen fibers were reduced by cotreatment with NAC and PLZ (Fig. 6e). The IL-6-positive area index was also decreased in the cotreatment group. However, in cotreatment groups, expression of PPARγ was increased significantly and oxidative stress markers HMOX-1 and Nrf2 were decreased compared with the control group (Fig. 6e). To assess the toxicity and safety of this combination therapy, we monitored the weight change of each mouse and the average food intake of each group. Although there was no statistical difference in the average weight of each group, mice in the cotreatment group showed a weight gain trend (Fig. 6d, Supplementary Fig. S11B). The average food intake by mice in the cotreatment group showed no significant difference from the control group (Supplementary Fig. S11D). Nevertheless, mice in the GEM group showed significant loss of appetite and weight gain cessation because of drug side-effects. Thus, NAC + PLZ co-treatment has excellent potential as a new approach to inhibit tumor growth.

**Fig. 7:**
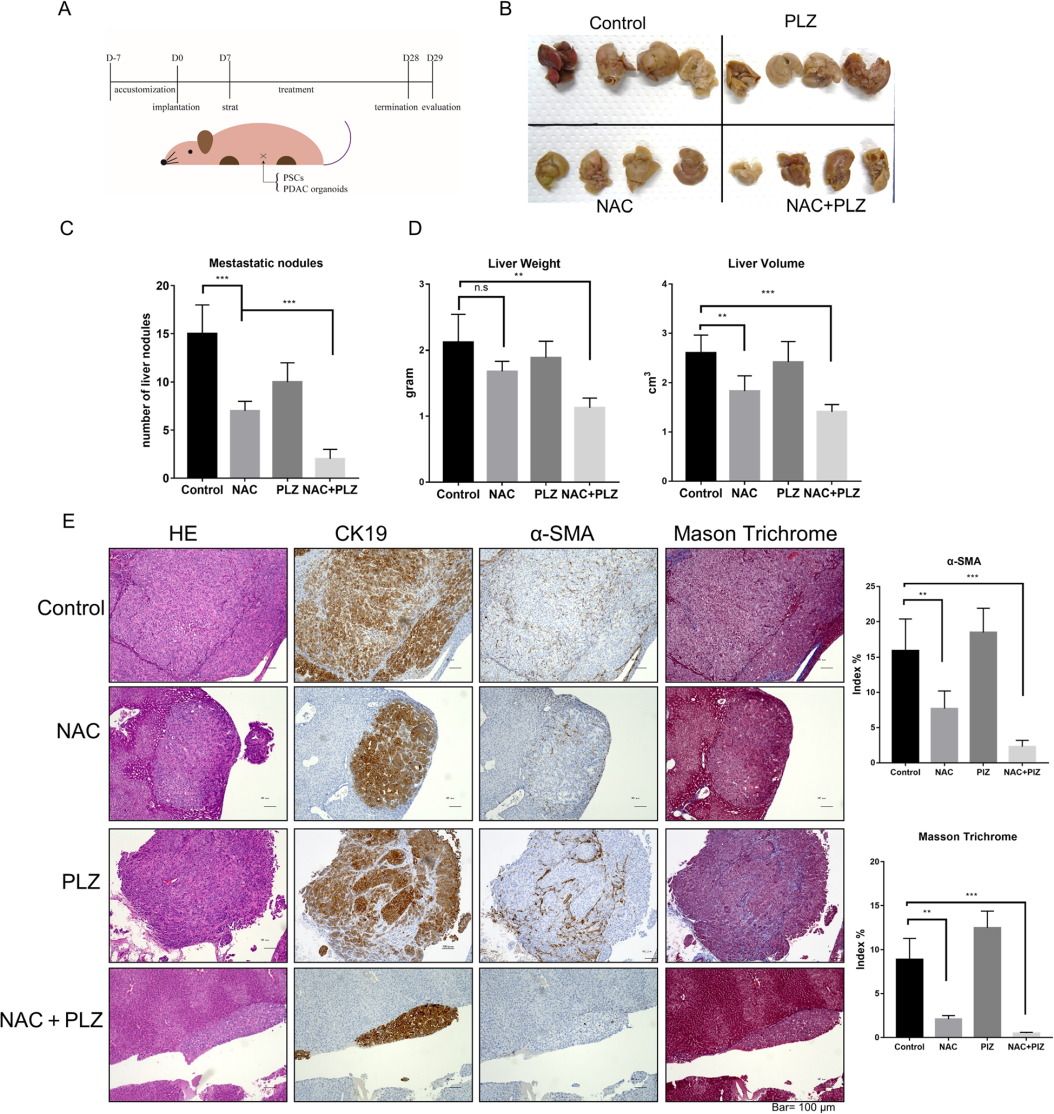
Cotreatment with NAC and PLZ decreased liver metastasis in the xenografted organoid model with PSC co-implantation. **a** The spleens of nude mice were implanted with KPC mouse-derived organoids (1 × 10^5^) and mouse PSCs (1 × 10^5^). After 1 week, mice were intravenously injected with vehicle (100 μl PBS + 0.1 μl DMSO, *n* = 5), NAC (500 mg/kg, n = 5), PLZ (4 mg/kg, n = 5), or their combination (n = 5) once every 2 days for 21 days. On day 28, we sacrificed the mice and evaluated their hepatic metastasis. **b** Representative photographs showed the number of disseminated visible nodules in the liver, and liver volume was decreased after combined treatment with NAC and PLZ. **c**-**d** The metastasis nodules (**c**) and the tumor weight and volume (**d**) in mice liver were evaluated. **e** H&E staining, Masson’s staining trichrome and Immunohistochemical staining of α-SMA and CK19 were performed. *P < 0.05; **P < 0.01; ***P < 0.001; n.s, no significance

Next, we co-implanted KPC organoids with PSCs into the spleen of nude mice to investigate the effects of co-treatment on tumor metastasis. Mice were treated intraperitoneally with the vehicle, NAC, PLZ, or a combination for 3 weeks. On day 28, the mice were sacrificed, and their liver metastases were harvested and evaluated (Fig. 7a-c). Compared with control, the number of metastatic nodules (15 vs. two, average) in the liver (Fig. 7d) (liver volume: 2.61 vs. 1.41 cm^3^, average; liver weight: 2.12 vs. 1.13 g, average; Fig. 7e) was decreased significantly in the combined treatment group, although NAC or PLZ alone also decreased the number of metastatic nodules (Fig. 7d). The number of peritoneal disseminated nodules was also significantly decreased after the combined treatment (Supplementary Fig.S11E-G). We also performed immunohistochemical staining to evaluate metastasis nodules. NAC and NAC + PLZ groups showed significant decreases in α-SMA-positive areas, but metastasis nodules in the latter group showed smaller areas (Fig. 7e). Pathological studies on liver slices showed that the NAC + PLZ had not produced significant liver damage, which safety was furtherly confirmed. These findings indicate that NAC suppresses PDAC organoids’ metastasis, and combined treatment of NAC with PLZ has cooperative effects on inhibiting tumor metastasis.

## Discussion

In this study, we investigated the effects of NAC on PCCs and PSCs and its functional effect on pancreatic cancer-stroma interactions. We found that PCCs efficiently increased the cell viability, migration, and invasion of PSCs. PSCs also increased these abilities of PCCs. Through these tumor-stroma interactions, PCCs showed more rapid growth and higher malignancy. However, NAC strongly suppressed the activities of PSCs, decreased the production of growth factors and reduced cell oxidative stress level at a low concentration rather than those of PCCs and attenuated the protumorigenic effects promoted by cancer-stroma interactions. NAC-treated PSCs were reactivated after exposure to PCC-SN, but co-treatment with NAC and PLZ maintained their quiescent-like state. Thus, NAC enhanced the therapeutic effect of PLZ at low concentrations in PCCs. This is the first report showing that NAC-induced quiescent-like PSCs from an activated state was maintained by combined treatment with PLZ. This new combination therapy may induce conversion from protumorigenic PSC to anti-tumorigenic PSCs, which leads to remodeling the tumor microenvironment of pancreatic cancer.

Current studies have focused mainly on suppressing pancreatic cancer desmoplasia or disrupting cancer-stroma interactions. Previously, agents such as Pirfenidone and calpeptin have been used to suppress desmoplasia, making PCCs more sensitive to Gemcitabine [16, 17]. Moreover, Sujit, et al. combined Pirfenidone and NAC to decrease tumor growth in orthotopic tumor models via inhibition of TGF-β and ROS in PSCs [19]. Thus, anti-fibrotic and anti-NAF therapeutic strategies appear to have a significant effect in anti-tumor therapy. However, the exact mechanism underlying their combined effect and the functional or state alteration of NAC-treated PSCs were yet to be investigated. In the present study, NAC treatment inhibited activated PSCs and significantly decreased the growth factors and prominent extracellular matrix (ECM) via alterations of multiple pathways. Our data also revealed that NAC not only decreased desmoplasia but also induced a quiescent-like state in PSCs. Quiescent or resting PSCs are star-shaped stellate cells that are activated when needed [34]. After external stimulation such as oxidative stress, ROS, chemokines or growth factors, quiescent fibroblasts can reversibly transfer to normally activated fibroblasts (NAFs), which express α-SMA and Vimentin and present spindle-shaped morphology [34]. These fibroblasts show high expression of α-SMA, which is why they are also called myofibroblasts [35]. However, upon encountering chronic stimuli, activated PSCs gain enhanced proliferative properties that induce a fibrotic tumor microenvironment. Consistent with previous studies, we observed PSCs’ morphological changes after NAC treatment and decreased oxidative stress level and expression of α-SMA. Other antioxidants like BHA have also decreased the oxidative stress level in PSCs and decreased expression of α-SMA, thus, these effects were seemly dependent on intracellular GSH levels, but NAC showed more superiority than BHA. Moreover, we found changes in signaling pathways of NAC-treated PSCs. These data also indicate that NAC altered the activated state of PSCs to a quiescent-like state. However, further study is needed to investigate whether quiescent PSCs induced by NAC are maintained when PSCs encounter continuous stimuli from PCCs in vivo.

It has been well reported that multiple signaling pathways and molecules are involved in the activation and pro-tumor cell functions of PSCs, including PI3k-AKT, NF-κB, and ERK1/2 signaling pathways [36–40]. The change of these signaling pathways underlying NAC-treated PSCs was also investigated in this study. We found that PCCs increased activation of PI3K-AKT and NF-κB signaling pathways in PSCs derived from tumor tissue via cancer-stroma interactions. The present data also revealed that NAC treatment significantly suppressed activated PI3K-AKT and NF-κB pathways, but not of the ERK1/2 signaling pathway in PSCs. Moreover, we found that the expression of PPARγ was increased in PSCs treated with NAC and that co-treatment with NAC and PLZ, a ligand of PPARγ, was significantly inhibited PI3K-AKT and NF-κB pathways, but also activation of ERK1/2 in PSCs. The expression level of PPARγ decreases markedly during activation of hepatic stellate cells [25]. Our knockdown experiments also confirmed that siPPARγ affected the effects of NAC in decreasing the activity of PSCs. These findings suggested when the expression of PPARγ was increased in quiescent-like state PSCs induced by NAC, administration of PPARγ ligand, like PLZ, seemly to be feasible to maintain this state.

Previously, PLZ was often used for type II diabetes but recently identified as a potential therapeutic agent to inhibit proliferation and metastasis of PCCs [41]. However, the treatment of a high concentration of PLZ was reported to increase bladder cancer [42]. In this study, we administrated PLZ with NAC at a safe concentration in co-culture or implantation of PSCs and PCCs and observed promising therapeutic effects. These data suggest that NAC increases sensitivity to PLZ in PCCs, although it cannot inhibit PCCs directly. Thus, co-treatment with NAC and PLZ may exert its therapeutic effects in both PSCs and PCCs.

We found that NAC inhibited most ECM proteins’ secretion, but the expression of collagen type IV, one of the major components of the basement membrane (BM), was maintained even in NAC-treated PSCs. BM is a sheet-like structure that separates ductal epithelial cells from the surrounding stroma. PCCs also increase the secretion of various Matrix metalloproteinases (MMP) to decompose the BM structure for their invasion [43]. .The BM primarily consists of collagen IV degraded by extracellular proteases such as MMP2 and MMP9 [44]. We previously reported that PSCs secreted MMP2 and induced BM destruction in the cancer-stromal microenvironment [24]. The present results showed that NAC decreased the expression of MMP2 in PSCs, which possibly maintained the BM structure and impeded PCCs from invading into the stroma. These data indicate that the microenvironment in pancreatic cancer was altered to an anti-tumor microenvironment by NAC treatment. To clarify the precise mechanism induced by the changes in collagen type IV and MMP-2 expression when PSCs were treated with NAC, further studies will be performed in the future.

Tumor organoid is considered as a brand-new model tool in biomedical research. In a previous study, we demonstrated that an ERK inhibitor reduces the number of metastasis in xenografts formed by tumor organoids [45]. In the present study, we performed in vivo xenograft experiments using KPC mouse-derived cancer organoids representing the microenvironment in PDAC, including PSCs. PSCs co-cultured with organoids from pancreatic cancer-induced excessive desmoplasia compared with co-culture with PCCs, which indicated that this organoid model is of great benefit to investigate the therapeutic effect of combined NAC and PLZ treatment. By this model, the effects of co-treatment therapy were confirmed in both subcutaneous tumor growth and splenic liver metastasis, as well as safety advantages.

## Conclusion

In summary, the present data indicated that NAC treatment decreases the activity of PSCs and attenuates cancer-stroma interactions. Moreover, the combination of NAC and PLZ maintains the quiescent-like state of PSCs, which leads to an enhanced therapeutic effect on tumor stromal components. Furthermore, NAC makes PCCs more sensitive to PLZ treatment. Taken together, the present data suggest that combination therapy of NAC and PLZ is a promising treatment that targets both stromal and cancer cells in pancreatic cancer.

## Supplementary Information

**Additional file 1: Supplementary Table S1.** Primers and their sequences used for qRT-PCR.

**Additional file 2: Supplementary Table S2.** Primary and secondary antibodies used for western blotting and immunofluorescence.

**Additional file 3: Supplementary Figure S1.** Analyses of α-SMA and CK19 immunofluorescence staining in SUIT-2 or PSCs isolated from PDAC patients. PSCs presented a fibroblast-like appearance and expressed α-SMA, but not CK19 (original magnification: × 200). Pancreatic cancer cell (PCCs) line SUIT-2 expressed CK19, but not α-SMA. **Supplementary Figure S2.** Toxicity effects of NAC to PSCs and PCCs. (A-B) IC_50_ values were measured in PSC1–3 and PCCs by determined by CellTiter-Glo luminescent cell viability assay after 72 h treatment with indicated NAC concentrations. (C) Representative microscopy time-lapse images of PSC1 and PSC2 cells treated with 6 mM NAC in which concentration toxic to PSCs. (D) (Left) PSC1 and PSC2 cells were treated with 2.5 mM NAC for 48 h, stained with annexin V-FITC/PI, and then analyzed by flow cytometry. (Right) Quantification of apoptotic cells induced by NAC treatment. (E) the expression of cleaved caspase-3, a marker of apoptosis, was determined by western blotting in PSCs. PSCs were treated with PBS (as control), 2.5 mM NAC, or 20μM H_2_O_2_ (positive control). (F) the expression of α-SMA, an active marker of PSC, was determined by western blotting in PSC2, which treated with different concertation of NAC with or without supernatant from SUIT-2 (SUIT-SN). (G) Migration of PSC2 after 1 mM NAC treatment with or without SUIT-SN was analyzed. Migrated cell numbers were normalized by total cell numbers. H&E staining; original magnification, × 100. Scale bar = 100 μm. (H-I) Migration and invasion assays of PSCs and PCCs from Fig. 1c-d. Graphs show numbers of cells calculated from five fields. The exact number of migrated cells was counted. Scale bar = 100 μm. H&E staining; original magnification, × 100. (J) Effects of NAC were performed in PCCs and PSCs on mRNA expression of Nrf2, HMOX-1, NQO1, and GCLC. PBS was used as Control. *P < 0.05; **P < 0.01; ***P < 0.001; n.s, no significance. **Supplementary Figure S3.** NAC decreases the activation of PSCs and induces a quiescent-like state. (A) Lipid droplet accumulation assay of untreated or NAC-treated PSC1 and PSC2 cells. Scale bar = 100 μm. (B) Representative morphological microphotograph of NAC-treated PSCs. Magnified microphotograph shows that treated-PSCs had a star-like morphology. Scale bar = 100 μm. (C) Cell viability assays of PSCs and NAC-treated PSCs for 72 h. (D) Migration assays of PSCs and NAC-treated PSCs. Migration values were normalized by the total cell numbers of each cell. H&E staining; original magnification, × 100. Scale bar = 100 μm. (E) Representative microphotograph of α-SMA (red) and Vimentin (green) immunofluorescence staining and nuclear (DAPI; blue) counterstaining in PSC2 cells on day 0 (Control) and day 28 (NAC-treated). Scale bar = 100 μm. (F) Glutathione (GSH) and Intracellular ROS level in PSC1 cells treated by 50 μM BHA with or without SUIT-CM. The values of RFUs (ROS level) were normalized to Control in each PSCs. (G) Western blotting of α-SMA and PPARγ of PSCs treated with 50 μM BHA or 2.5 mM NAC. *P < 0.05; **P < 0.01; ***P < 0.001; n.s, no significance. **Supplementary Figure S4.** NAC attenuates cancer-stroma interaction in pancreatic cancer. (A-B) Representative graphs of migration and invasion assays of PSCs (A) and PCCs(B) from Fig. 3c and f. H&E staining; original magnification, × 100. Scale bar = 100 μm. (C) Effects of NAC in PCCs which induced by PSCs in the co-culture system. PSCs were first seeded in the plate and treated with NAC or PBS (Control). After 24 h, PCCs were seeded in upper Chambers (8 μm), and the numbers of migrated or invaded PCCs were evaluated after incubation for 24 h or 48 h. Migrated and invaded cells were counted. H&E staining; original magnification, × 100. Scale bar = 100 μm. **Supplementary Figure S5.** NAC inhibited IL6-STAT3 signal between stromal-cancer interactions in pancreatic cancer. (A) Western blotting of the p-STAT3 level of SUIT-2 and PANC-1 cells after treatment with 20 μM CTS. Activation of STAT3 was inhibited by Cryptotanshinone (CTS) treatments. (B) Western blotting of p-STAT3, STAT3, c-Myc and P21 level of SUIT-2 and PANC-1 cell after indicated agent treatment. Agents were as followed: SN from PSC (PSC-SN), 5 μg/ml IL-6 neutralizing antibody (IL6 neu), 20 ng/ml human recombination IL-6 (hr IL-6), SN from NAC-treated PSC (NAC-PSC-SN). (C) Representative graphs of migration and invasion assays of SUIT-2 from Fig. 3i. (D) Migration and invasion assays of PANC-1 cells were performed at 24 h or 48 h after treatment with indicated agents. Migrated and invaded cells were counted. (E) Invasion assays of SUIT-2 cells in collagen type I, type IV or a normal Martrix coated transwell chamber. Graphs show the number of cells calculated from five fields randomly. H&E staining; original magnification, × 100. Scale bar = 100 μm. **Supplementary Figure S6.** PPARγ mediated NAC induced attenuation effects in PSCs. PSC1 cells and NAC-treated PSC1 cells were transfected with si-control or si-PPARγ (si1 and si2). (A) Western blotting of PPARγ and α-SMA level of PSC1 and NAC-treated PSC1. (B) Cell viability assays of PSC1 and NAC-treated PSC1 that transfected with siRNA. (C) Migration assays of PSCs and NAC-treated PSCs that transfected with siRNA. Migration values were normalized by the total cell numbers of each cell. H&E staining; original magnification, × 100. Scale bar = 100 μm. **Supplementary Figure S7.** Effects of combined treatment of NAC and PLZ. (A) Western blotting of the indicated proteins in PSC1 and NAC-treated PSC1 cells with and without SUIT-SN treatment. (B) Microphotograph of PSC1 cells after treatment with NAC, PLZ, or both for 3 days. Scale bar = 100 μm. (C) Migration and invasion assays of PSC1 cells were performed after treatment with 20 μM PLZ or the Control (DMSO) for either 24 or 48 h. (D) Cell viability assays of PSC1 and PSC2 cells after treatment with the indicated agent. (E) Migration and invasion assays of PANC-1 cells treated with the indicated agent, respectively. Migrated or invaded cell numbers were normalized by the total cell number of each cell. Original magnification, × 100. Scale bar = 100 μm. *P < 0.05; **P < 0.01; ***P < 0.001; n.s, no significance. **Supplementary Figure S8.** Correlation between the expression of PPARγ and oxidative stress response. (A) Correlation analysis between the expression of PPARγ and other genes related to oxidative stress at mRNA level by TCGA public database. RNA seq data of 184 total samples from TCGA were analyzed by cbioportal (http://www.cbioportal.org/). Spearman scores and Pearson scores were listed. (B) Effects of NAC, PLZ and co-treatment were performed in PSCs on mRNA expression of PPARγ, PLIN1, FABP4, Adipo, CD36, LPL and RXRα. DMSO was used as control. **Supplementary Figure S9.** Inhibitory effect of NAC on subcutaneous tumor growth in vivo. (A) Nude mice (*n* = 12) were subcutaneously transplanted with only SUIT-2 cells (1 × 10^6^) into the left flank and together with PSC1 cells (5 × 10^5^, respectively) into the right flank. After 1 week, the mice were injected intraperitoneally with NAC (*n* = 6) or PBS (Control, n = 6) three times weekly for 4 weeks. (Bottom) Representative photograph showing significantly reduced tumor formation after 4 weeks of NAC treatment. (B) Tumor volume was significantly decreased in the NAC group after 4 weeks of treatment. (C) H&E staining, Masson’s staining trichrome and Immunohistochemical staining of α-SMA and PCNA. (D) Immunohistochemical staining of PPARγ, HMOX- 1 and Nrf2. (E) Compared with tumors in the left flank, Masson’s trichromatic staining showed much more intense in the right flank tumor, where the area of collagen fibers was significantly decreased by NAC treatment. (F) The right flank tumor showed a high rate of α-SMA-positive cells in the control group than the left side. The a-SMA-positive rate was increased on both sides by NAC treatment. (G) NAC decreased the PCNA index (%) in the right flank tumors, but not in the left flank’s tumors. **Supplementary Figure S10.** Co-culture KPC-derived organoids with PSCs replicated microenvironments in pancreatic cancer in vivo. (A) Scheme of 3D organoid versus 2D standard xenograft experiment. 1 × 10^5^ cancer cells or organoids derived from KPC mice were intra-splenically implanted into nude mice (*n* = 5/ group) with 1 × 10^5^ PSCs. After 3 weeks, we sacrificed the mice and evaluated their hepatic metastasis. Mouse liver metastases were made into paraffin sections and assessed by immunohistochemical staining of CK19 (B), α-SMA and Masson’s trichrome staining (B-C). **Supplementary Figure S11.** Cotreatment with NAC and PLZ inhibited subcutaneous tumor growth and liver metastasis in the xenografted organoid model with PSC co-implantation. (A) Representative photographs of mice when sacrificed in Fig. 6 treated with indicated reagents. (B) The weight change of each mouse from each group was recorded. (C) liver of mice from three groups was resected and analyzed drug toxicity and safety. (D) Average daily weight of food intake by each mouse in three groups. (E) Representative photographs of abdominal metastasis of mice in Fig. 7 treated with indicated reagents (arrow). (F) Disseminated visible nodules with a diameter larger than 1 mm are counted, NAC decreased the number of visible nodules, and combined treatment of NAC with PLZ further improved therapeutic efficacy. (G) Immunohistochemical staining of CK19 showed the amount of metastatic cell cluster. Original magnification, × 100. Scale bar = 100 μm. *P < 0.05; **P < 0.01; ***P < 0.001; n.s, no significance.

## Data Availability

All data generated or analyzed during this study are included either in this article or in the Additional file.

## Abbreviations

PSC: Pancreatic stellate cell
PDAC: Pancreatic ductal adenocarcinoma
PCC: pancreatic cancer cell
NAC: N-acetyl-cysteine
PPARγ: Peroxisome proliferator-activated receptor gamma
IC50: 50% inhibitory concentration
PLZ: Pioglitazone
KPC: LSL-Kras G12D/+; LSL-Trp53R172H/+; Pdx-1-Cre
ERK: Extracellular signal-regulated kinase
PI3K: Phosphatidylinositol-3 kinase
SN: supernatant
MMP: Matrix metalloproteinases
GSH: Glutathione
ROS: Reactive oxygen species
