# Supplementary material for: N-acetyl cysteine induces quiescent-like pancreatic stellate cells from an active state and attenuates cancer-stroma interactions
Source: J Exp Clin Cancer Res. 2021 Apr 15;40:133. doi: 10.1186/s13046-021-01939-1 (PMC8050903; doi:10.1186/s13046-021-01939-1)
Supplement: Supplementary file 3 — Additional file 3: Supplementary Figure S1. Analyses of α-SMA and CK19 immunofluorescence staining in SUIT-2 or PSCs isolated from PDAC patients. PSCs presented a fibroblast-like appearance and expressed α-SMA, but not CK19 (original magnification: × 200). Pancreatic cancer cell (PCCs) line SUIT-2 expressed CK19, but not α-SMA. Supplementary Figure S2. Toxicity effects of NAC to PSCs and PCCs. (A-B) IC50 values were measured in PSC1–3 and PCCs by determined by CellTiter-Glo luminescent cell viability assay after 72 h treatment with indicated NAC concentrations. (C) Representative microscopy time-lapse images of PSC1 and PSC2 cells treated with 6 mM NAC in which concentration toxic to PSCs. (D) (Left) PSC1 and PSC2 cells were treated with 2.5 mM NAC for 48 h, stained with annexin V-FITC/PI, and then analyzed by flow cytometry. (Right) Quantification of apoptotic cells induced by NAC treatment. (E) the expression of cleaved caspase-3, a marker of apoptosis, was determined by western blotting in PSCs. PSCs were treated with PBS (as control), 2.5 mM NAC, or 20μM H2O2 (positive control). (F) the expression of α-SMA, an active marker of PSC, was determined by western blotting in PSC2, which treated with different concertation of NAC with or without supernatant from SUIT-2 (SUIT-SN). (G) Migration of PSC2 after 1 mM NAC treatment with or without SUIT-SN was analyzed. Migrated cell numbers were normalized by total cell numbers. H&E staining; original magnification, × 100. Scale bar = 100 μm. (H-I) Migration and invasion assays of PSCs and PCCs from Fig. 1c-d. Graphs show numbers of cells calculated from five fields. The exact number of migrated cells was counted. Scale bar = 100 μm. H&E staining; original magnification, × 100. (J) Effects of NAC were performed in PCCs and PSCs on mRNA expression of Nrf2, HMOX-1, NQO1, and GCLC. PBS was used as Control. *P < 0.05; **P < 0.01; ***P < 0.001; n.s, no significance. Supplementary Figure S3. NAC decreases the activation of PSCs an [file 13046_2021_1939_MOESM3_ESM.pdf]

Figure S1

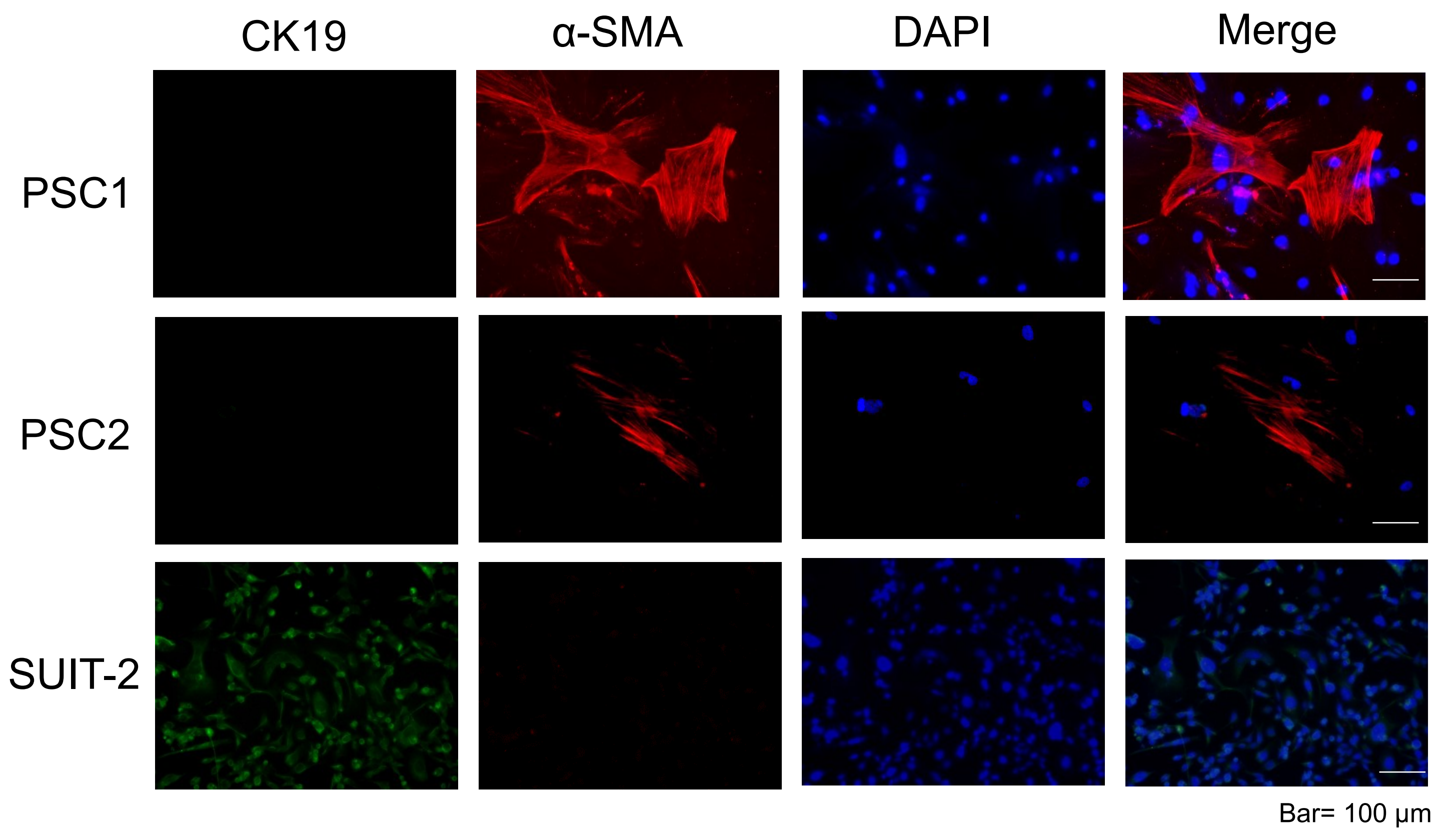

Figure S2

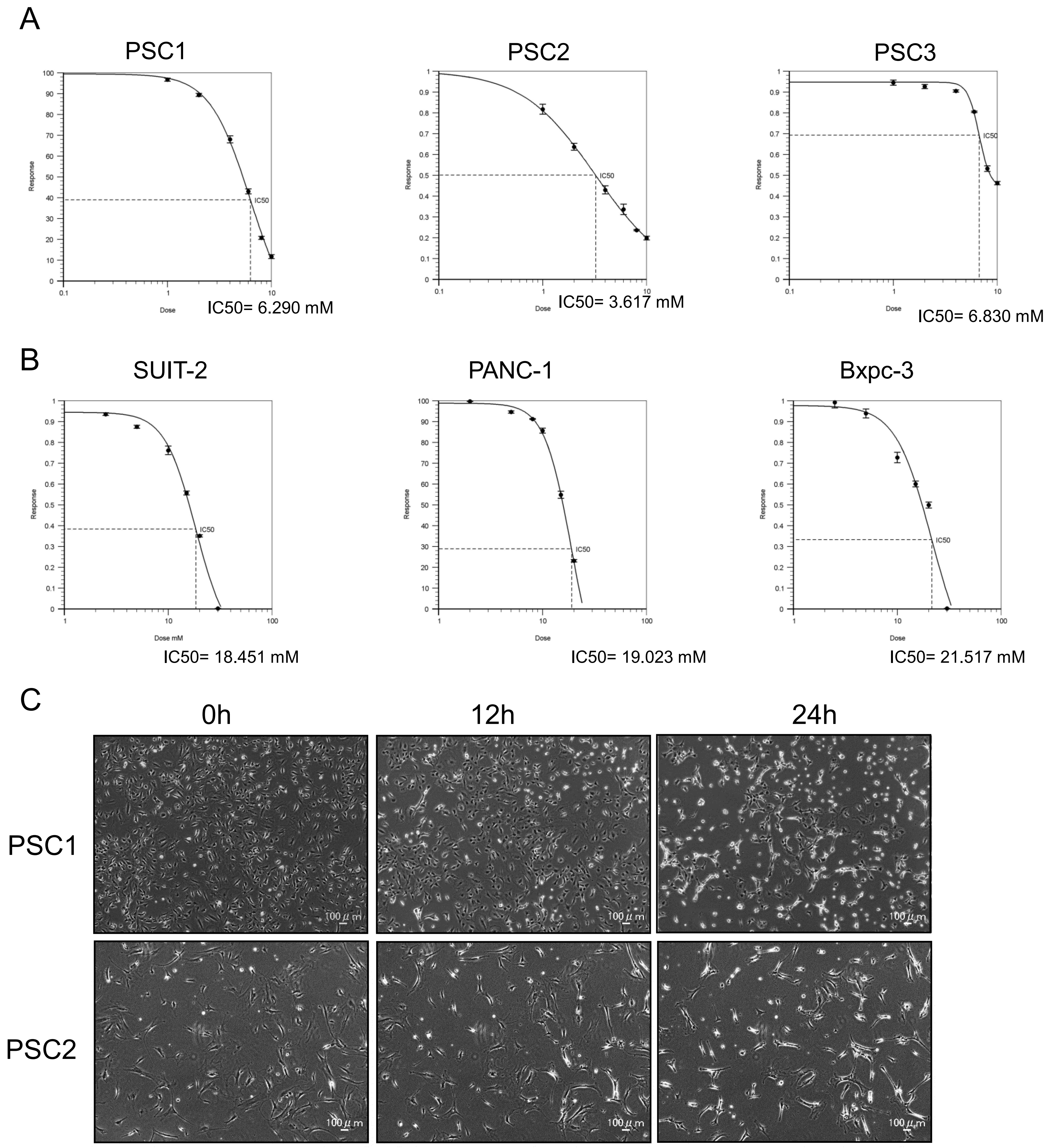
A
PSC1
IC50= 6.290 mM
PSC2
IC50= 3.617 mM
PSC3
IC50= 6.830 mM
B
SUIT-2
IC50= 18.451 mM
PANC-1
IC50= 19.023 mM
Bxpc-3
IC50= 21.517 mM
C
0h
12h
24h
PSC1
PSC2

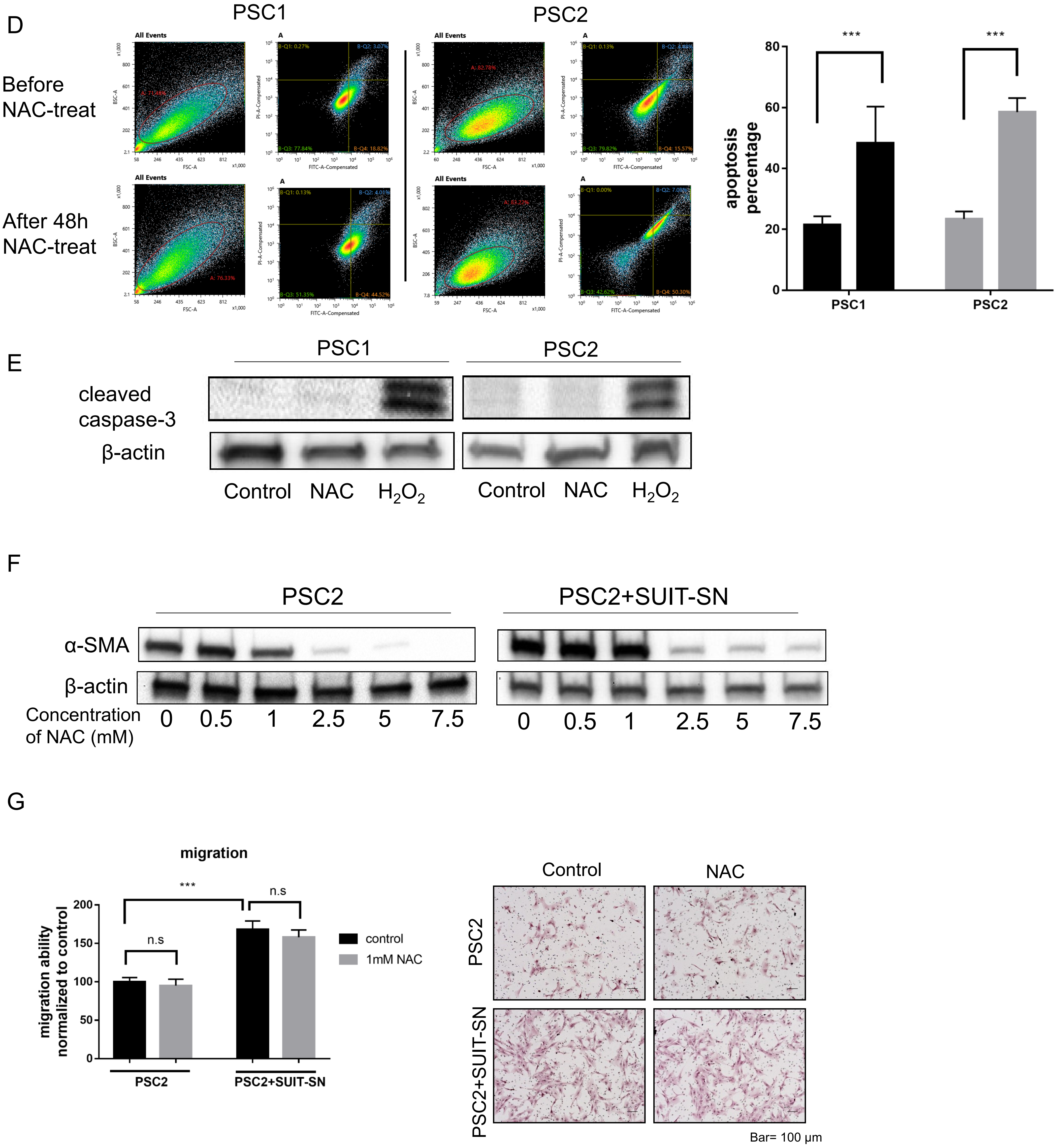
D
PSC1
PSC2
Before NAC-treat
After 48h NAC-treat
All Events
apoptosis percentage
***
***
80
60
40
20
0
PSC1
PSC2
E
PSC1
PSC2
cleaved caspase-3
β-actin
Control
NAC
H2O2
Control
NAC
H2O2
F
PSC2
PSC2+SUIT-SN
α-SMA
β-actin
Concentration of NAC (mM)
0
0.5
1
2.5
5
7.5
0
0.5
1
2.5
5
7.5
G
migration
migration ability normalized to control
***
n.s
n.s
200
150
100
50
0
control
1mM NAC
PSC2
PSC2+SUIT-SN
Control
NAC
PSC2
PSC2+SUIT-SN
Bar= 100 μm

H

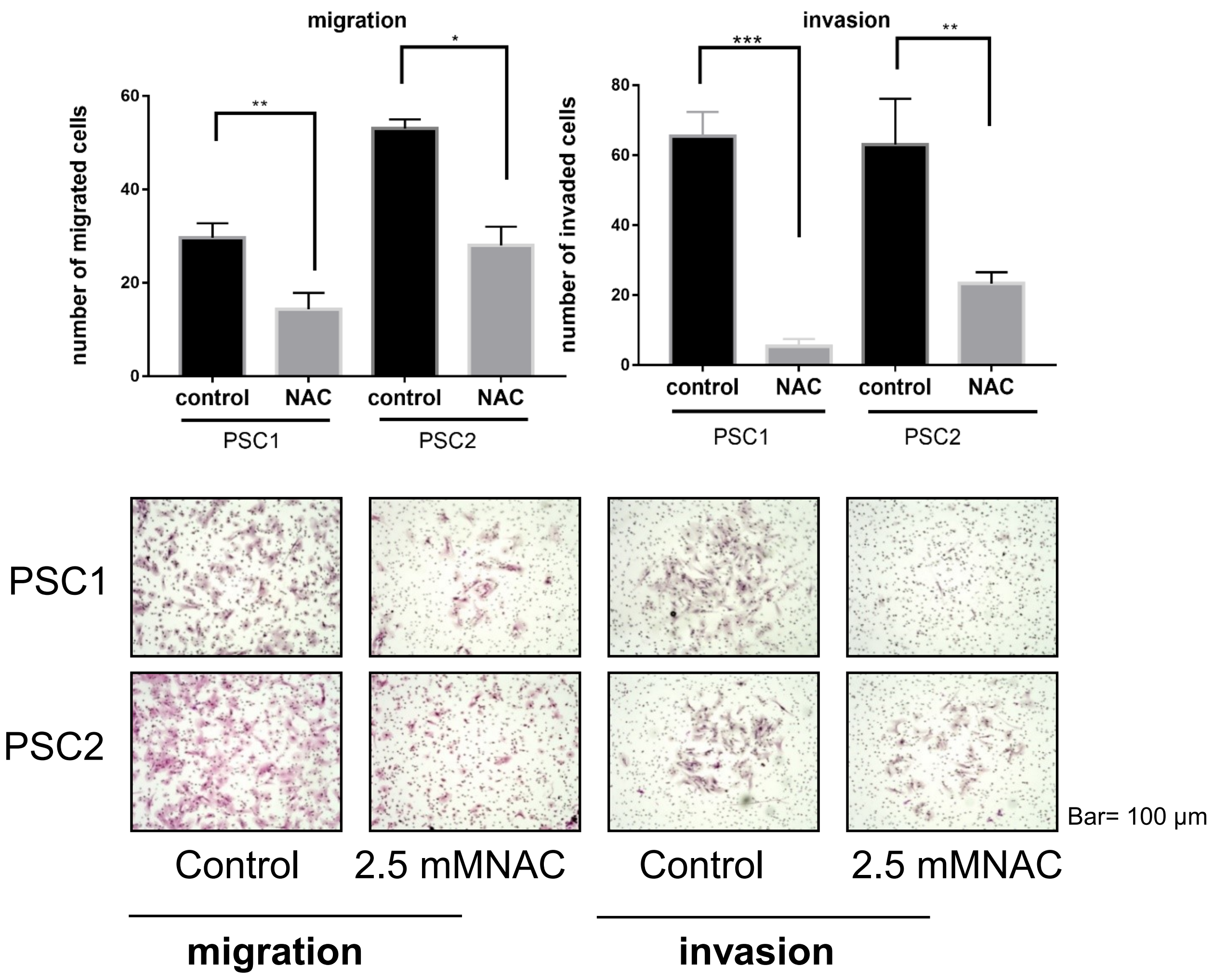
migration
invasion
number of migrated cells
number of invaded cells
control
NAC
PSC1
PSC2
PSC1
PSC2
Bar= 100 μm
Control
2.5 mMNAC
Control
2.5 mMNAC
migration
invasion

I

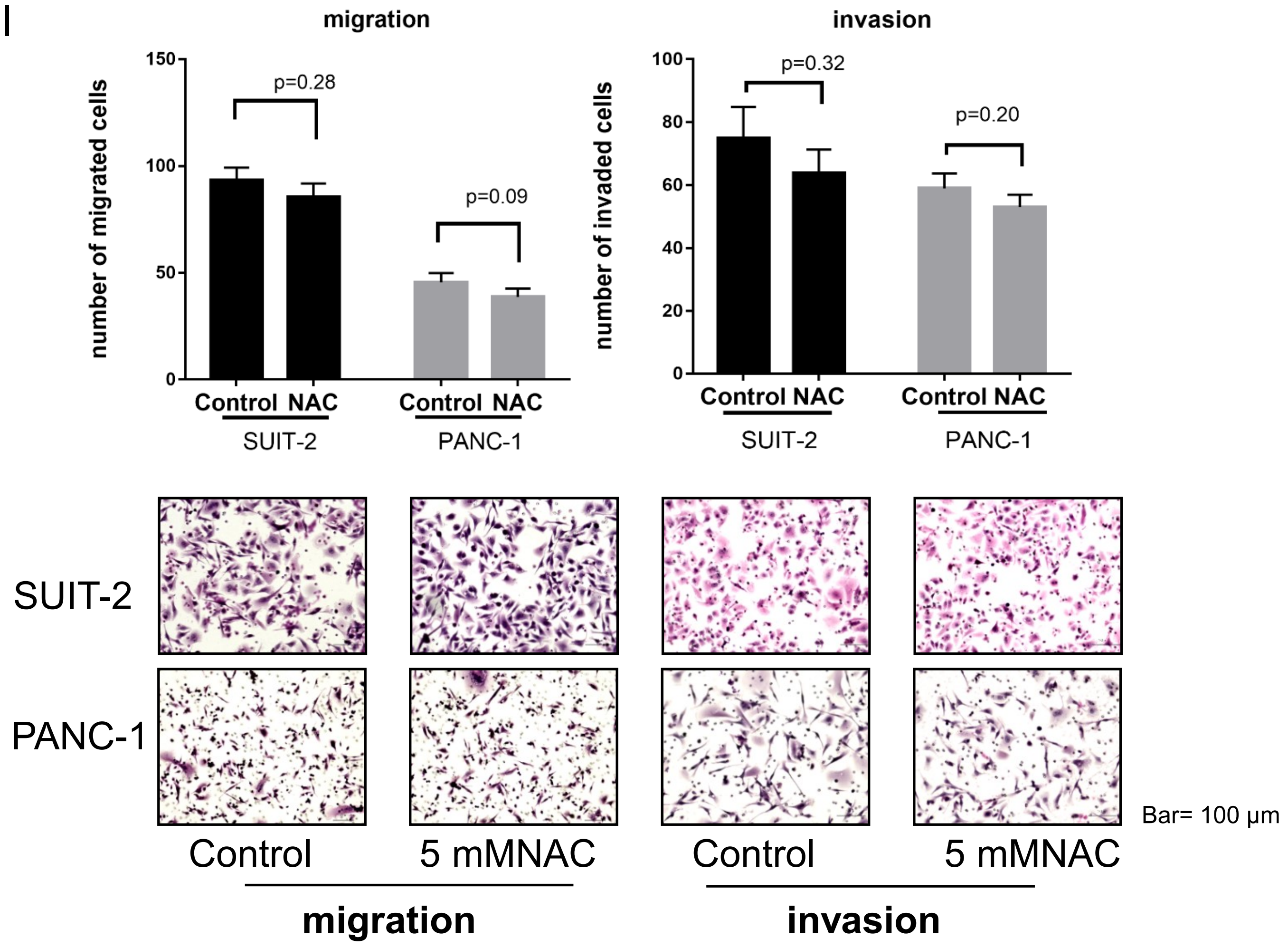
migration
invasion
number of migrated cells
number of invaded cells
p=0.28
p=0.09
p=0.32
p=0.20
Control NAC
SUIT-2
PANC-1
SUIT-2
PANC-1
Bar= 100 μm
Control
5 mMNAC
Control
5 mMNAC
migration
invasion

J

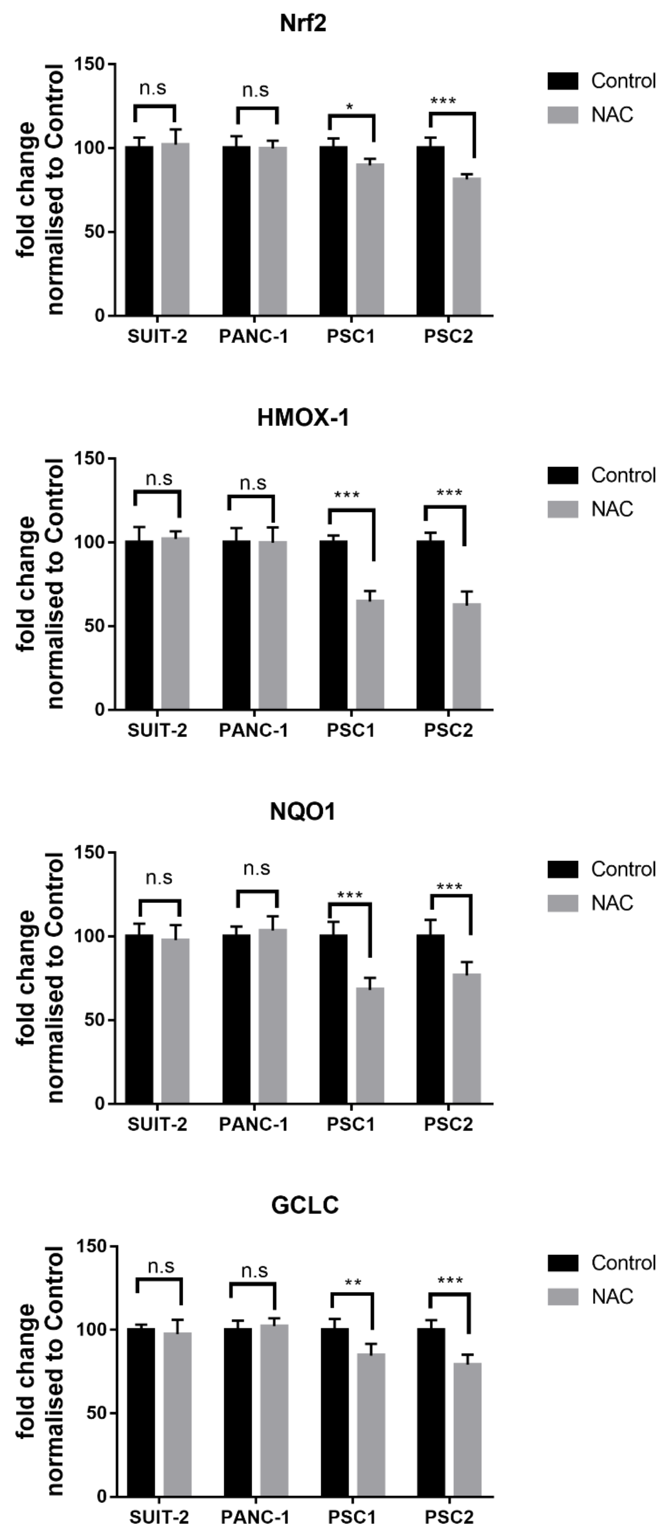
Nrf2
HMOX-1
NQO1
GCLC
fold change
normalised to Control
Control
NAC
n.s
SUIT-2
PANC-1
PSC1
PSC2

Figure S3

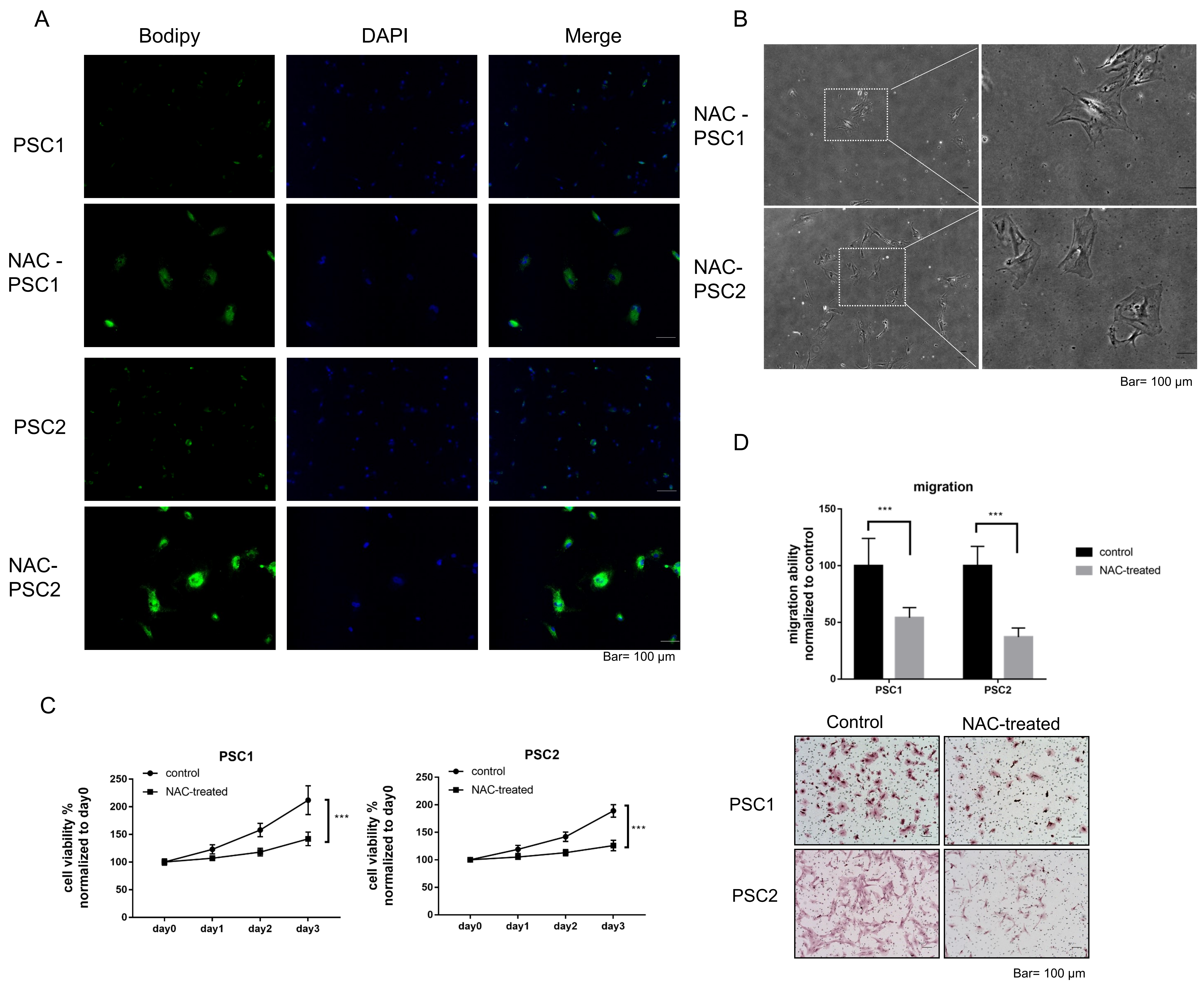
A
Bodipy
DAPI
Merge
PSC1
NAC - PSC1
PSC2
NAC- PSC2
Bar= 100 μm
B
NAC - PSC1
NAC- PSC2
Bar= 100 μm
C
PSC1
PSC2
control
NAC-treated
cell viability % normalized to day0
250
200
150
100
50
0
day0
day1
day2
day3
***
D
migration
migration ability normalized to control
150
100
50
0
***
PSC1
PSC2
control
NAC-treated
Control
NAC-treated
PSC1
PSC2
Bar= 100 μm

E

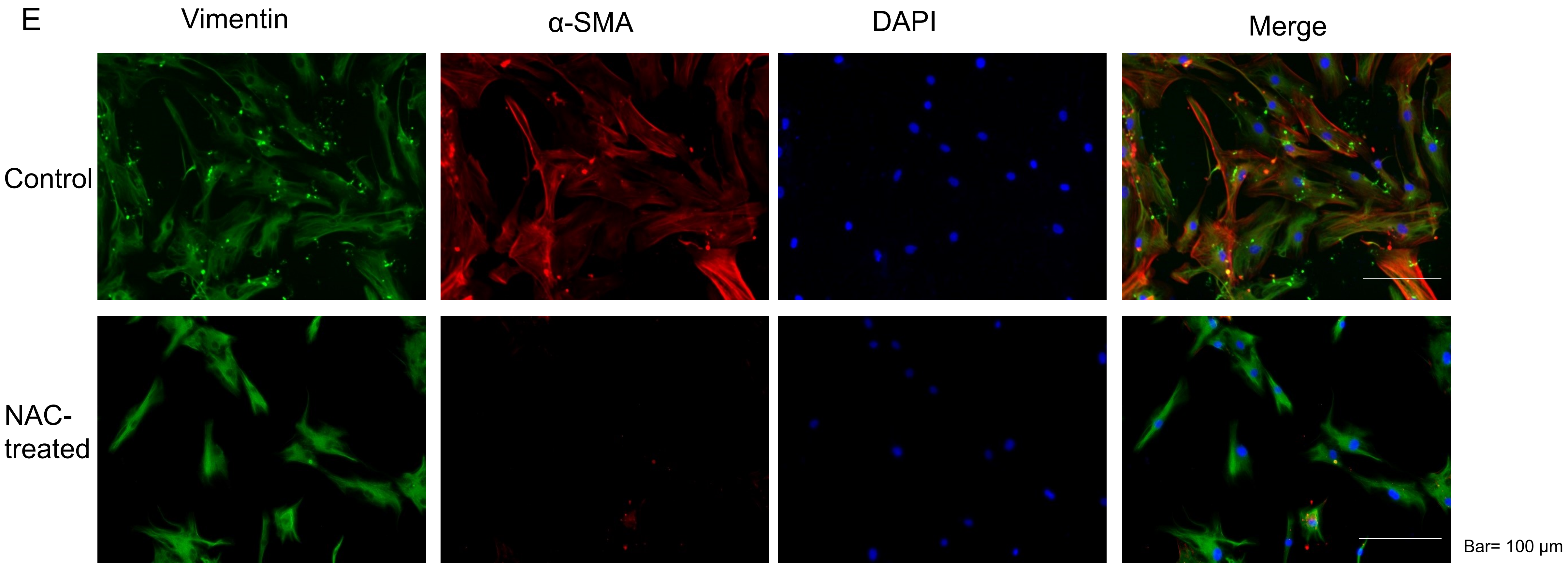
Vimentin
α-SMA
DAPI
Merge
Control
NAC-
treated
Bar= 100 μm

F

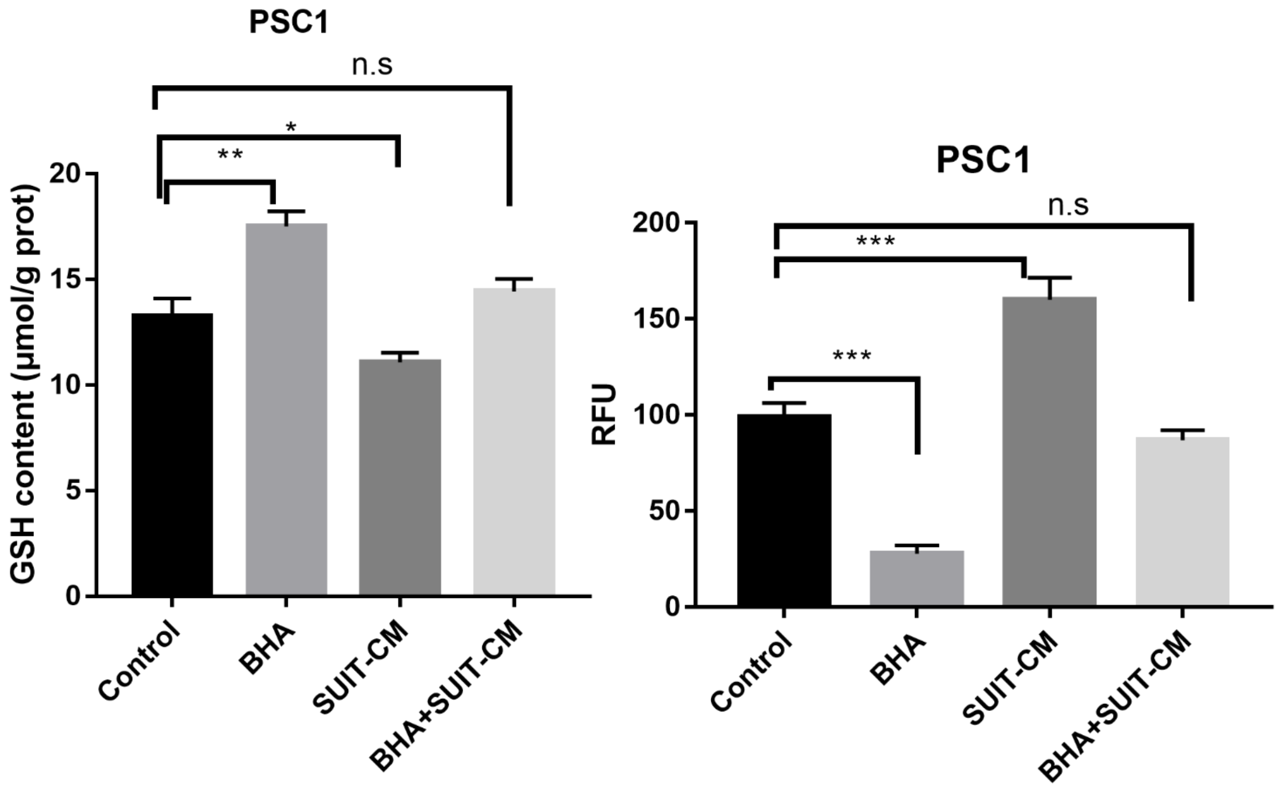
PSC1
n.s
*
**
GSH content (μmol/g prot)
20
15
10
5
0
Control
BHA
SUIT-CM
BHA+SUIT-CM
PSC1
n.s
***
***
RFU
200
150
100
50
0
Control
BHA
SUIT-CM
BHA+SUIT-CM

G

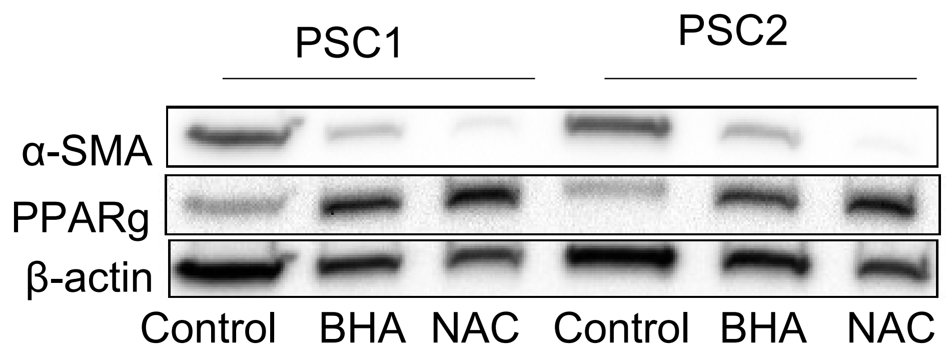
PSC1
PSC2
α-SMA
PPARg
β-actin
Control
BHA
NAC
Control
BHA
NAC

Figure S4

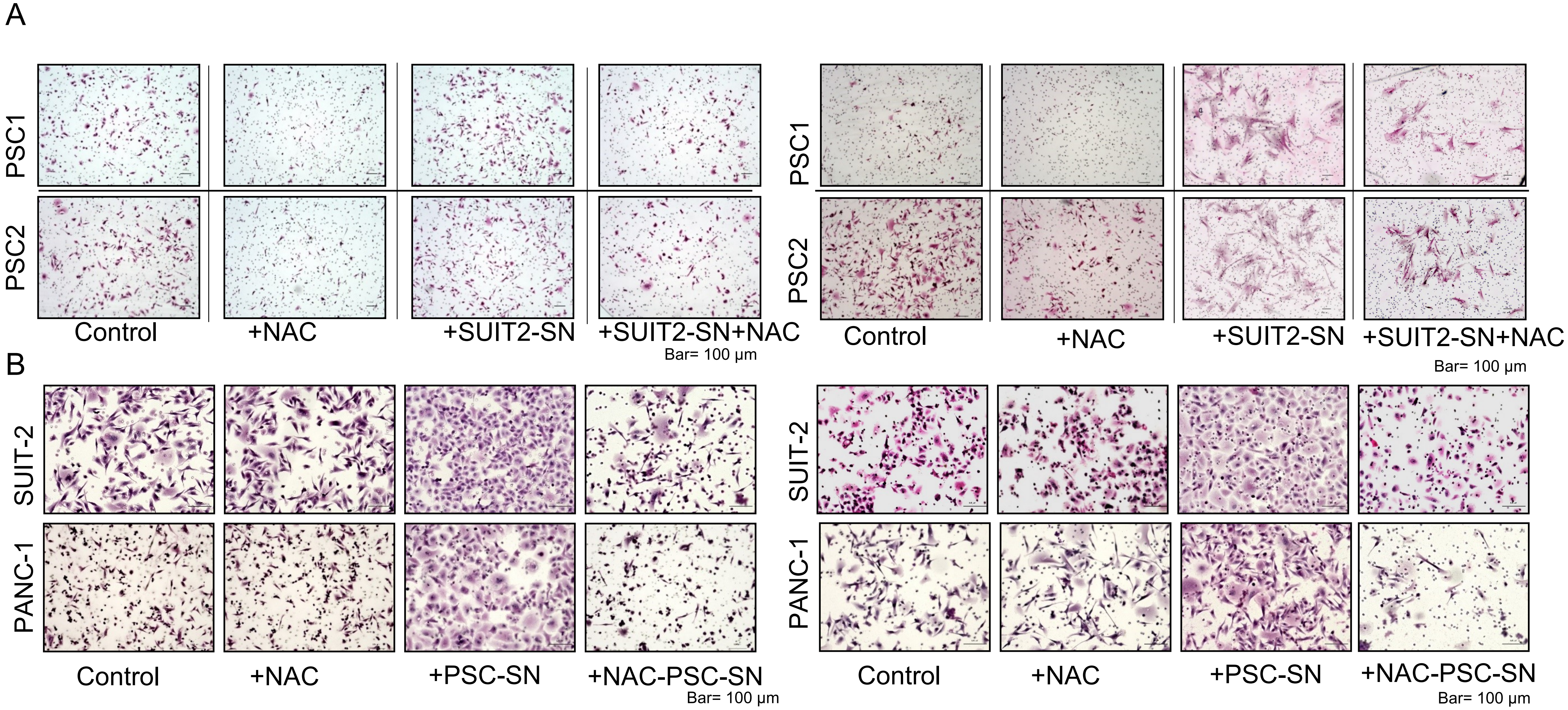
A
PSC1
PSC2
Control
+NAC
+SUIT2-SN
+SUIT2-SN+NAC
Bar= 100 µm
PSC1
PSC2
Control
+NAC
+SUIT2-SN
+SUIT2-SN+NAC
Bar= 100 µm
B
SUIT-2
PANC-1
Control
+NAC
+PSC-SN
+NAC-PSC-SN
Bar= 100 µm
SUIT-2
PANC-1
Control
+NAC
+PSC-SN
+NAC-PSC-SN
Bar= 100 µm

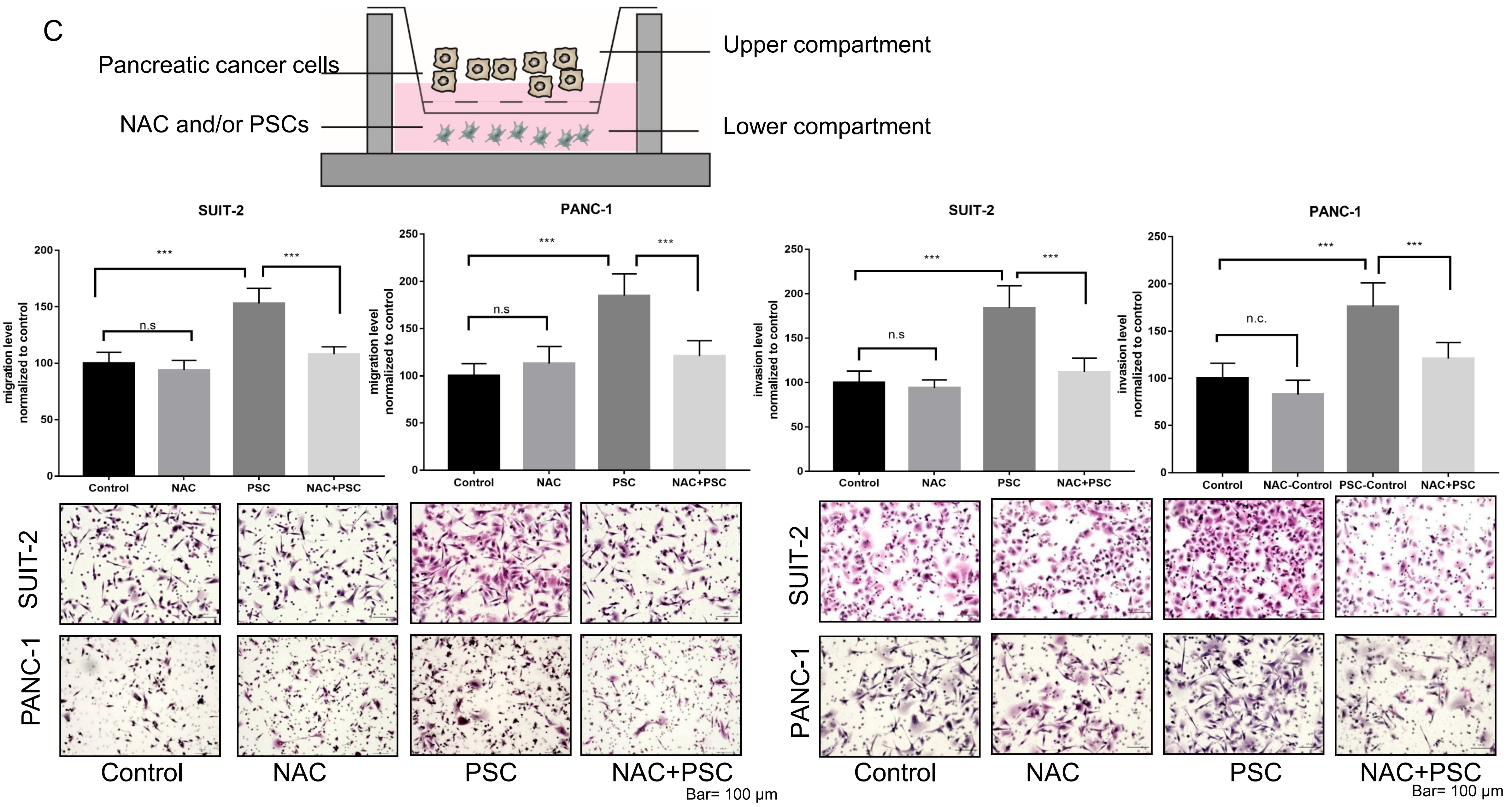
C
Pancreatic cancer cells
NAC and/or PSCs
Upper compartment
Lower compartment
SUIT-2
migration level normalized to control
Control
NAC
PSC
NAC+PSC
n.s
***
PANC-1
migration level normalized to control
SUIT-2
invasion level normalized to control
PANC-1
invasion level normalized to control
n.c.
NAC-Control
PSC-Control
SUIT-2
PANC-1
Bar= 100 μm

Figure S5

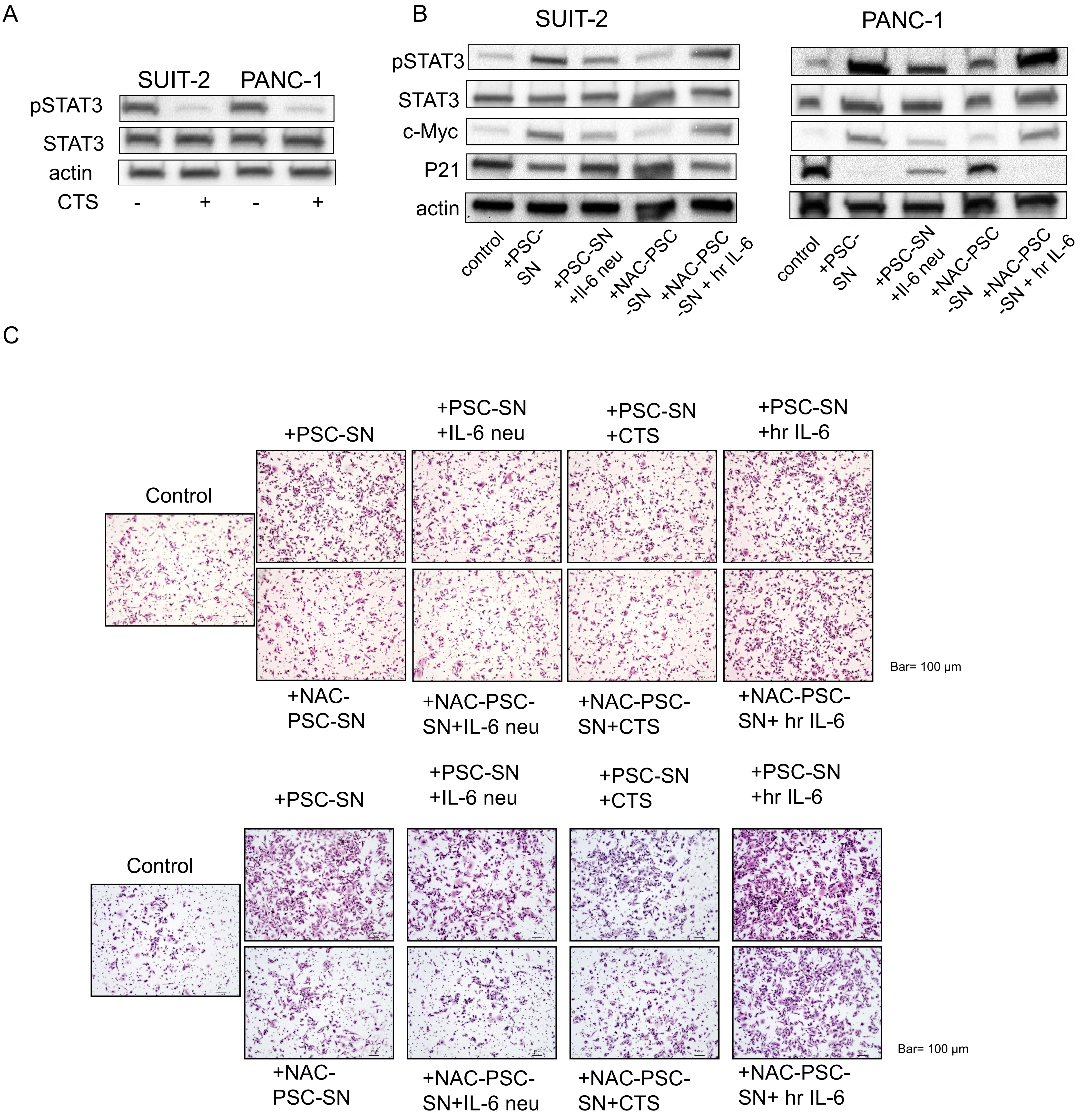
A
SUIT-2
PANC-1
pSTAT3
STAT3
actin
CTS
-
+
-
+
B
SUIT-2
PANC-1
pSTAT3
STAT3
c-Myc
P21
actin
control
+PSC-SN
+PSC-SN +Il-6 neu
+NAC-PSC-SN
+NAC-PSC-SN + hr IL-6
C
Control
+PSC-SN
+PSC-SN +IL-6 neu
+PSC-SN +CTS
+PSC-SN +hr IL-6
+NAC-PSC-SN
+NAC-PSC-SN+IL-6 neu
+NAC-PSC-SN+CTS
+NAC-PSC-SN+ hr IL-6
Bar= 100 µm
Control
+PSC-SN
+PSC-SN +IL-6 neu
+PSC-SN +CTS
+PSC-SN +hr IL-6
+NAC-PSC-SN
+NAC-PSC-SN+IL-6 neu
+NAC-PSC-SN+CTS
+NAC-PSC-SN+ hr IL-6
Bar= 100 µm

D

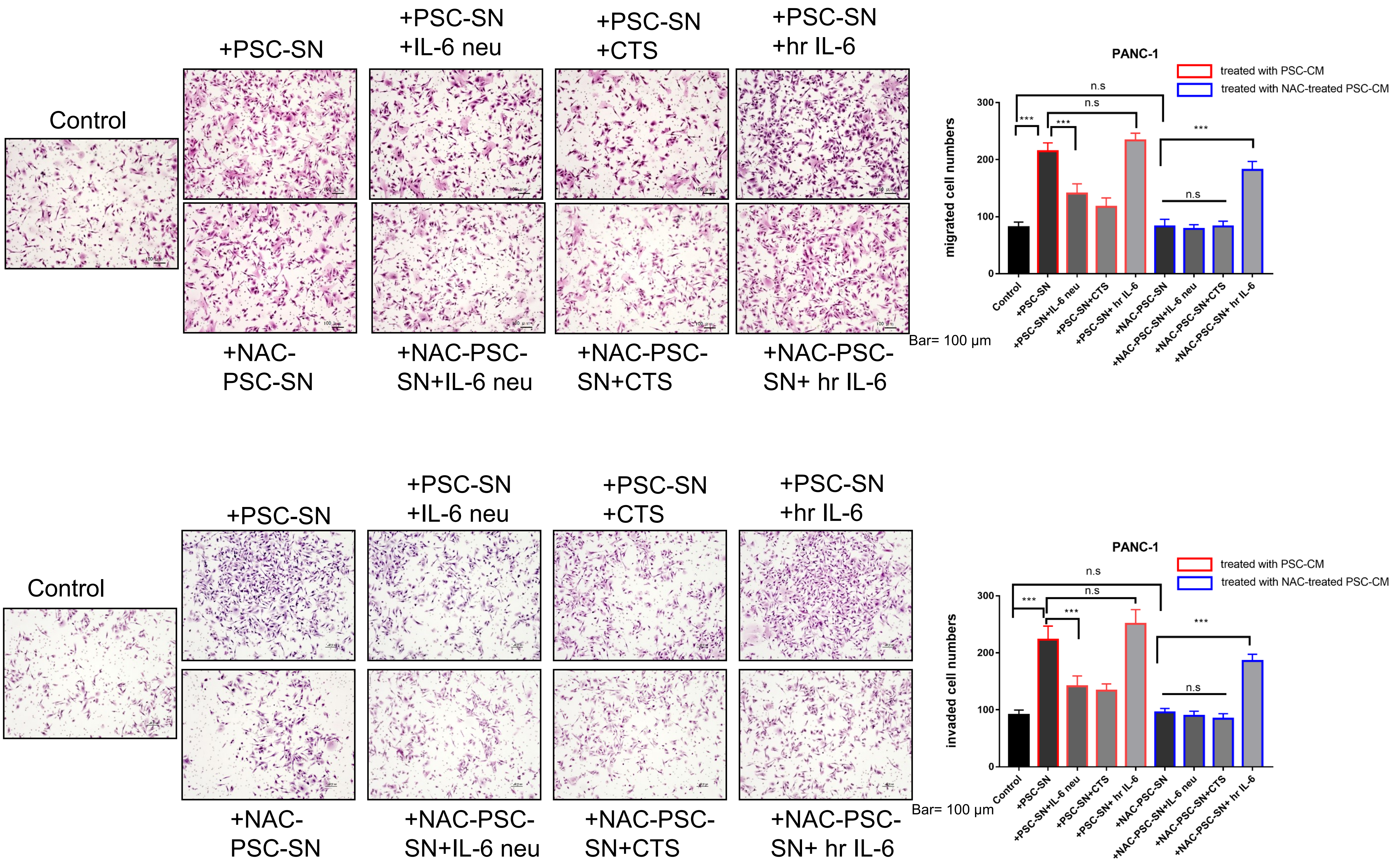
Control
+PSC-SN
+PSC-SN +IL-6 neu
+PSC-SN +CTS
+PSC-SN +hr IL-6
+NAC-PSC-SN
+NAC-PSC-SN+IL-6 neu
+NAC-PSC-SN+CTS
+NAC-PSC-SN+ hr IL-6
Bar= 100 μm
PANC-1
treated with PSC-CM
treated with NAC-treated PSC-CM
migrated cell numbers
n.s
***
0
100
200
300
Control
+PSC-SN
+PSC-SN+IL-6 neu
+PSC-SN+CTS
+PSC-SN+ hr IL-6
+NAC-PSC-SN
+NAC-PSC-SN+IL-6 neu
+NAC-PSC-SN+CTS
+NAC-PSC-SN+ hr IL-6
Control
+PSC-SN
+PSC-SN +IL-6 neu
+PSC-SN +CTS
+PSC-SN +hr IL-6
+NAC-PSC-SN
+NAC-PSC-SN+IL-6 neu
+NAC-PSC-SN+CTS
+NAC-PSC-SN+ hr IL-6
Bar= 100 μm
PANC-1
treated with PSC-CM
treated with NAC-treated PSC-CM
invaded cell numbers
n.s
***
0
100
200
300

E

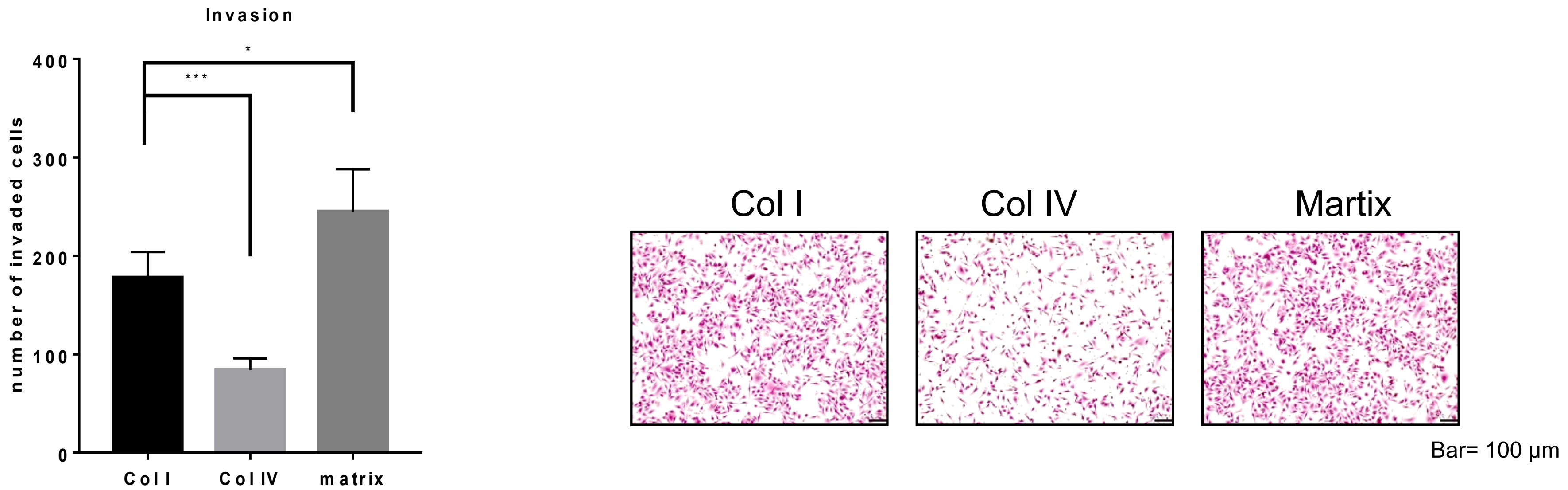
Invasion
number of invaded cells
0
100
200
300
400
***
*
Col I
Col IV
matrix
Col I
Col IV
Martix
Bar= 100 μm

Figure S6

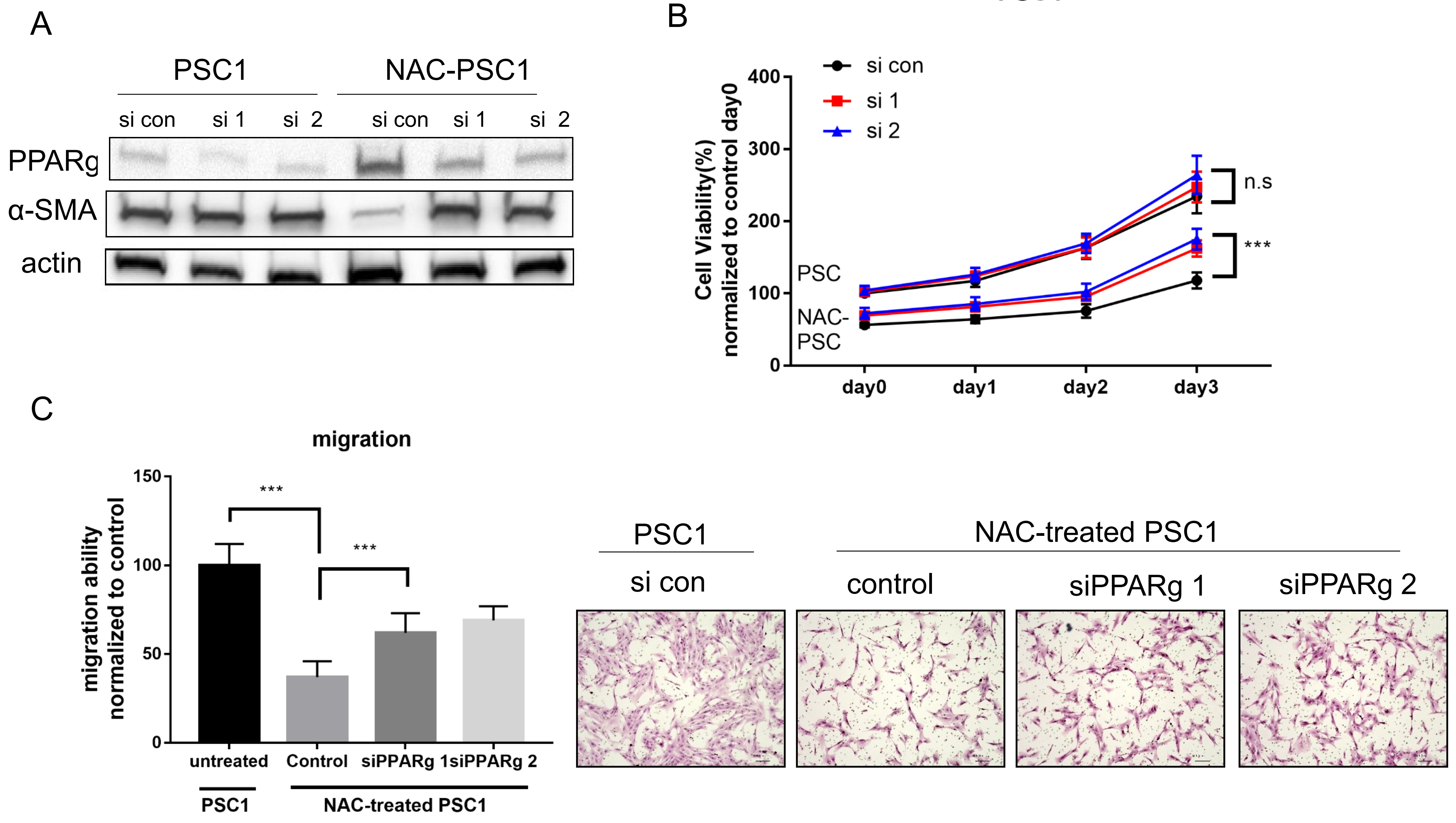
A
PSC1
NAC-PSC1
si con
si 1
si 2
si con
si 1
si 2
PPARg
α-SMA
actin
B
PSC1
si con
si 1
si 2
Cell Viability(%)
normalized to control day0
400
300
200
100
0
PSC
NAC-
PSC
n.s
***
day0
day1
day2
day3
C
migration
migration ability
normalized to control
150
100
50
0
***
***
untreated
Control
siPPARg 1
siPPARg 2
PSC1
NAC-treated PSC1
PSC1
NAC-treated PSC1
si con
control
siPPARg 1
siPPARg 2

Figure S7

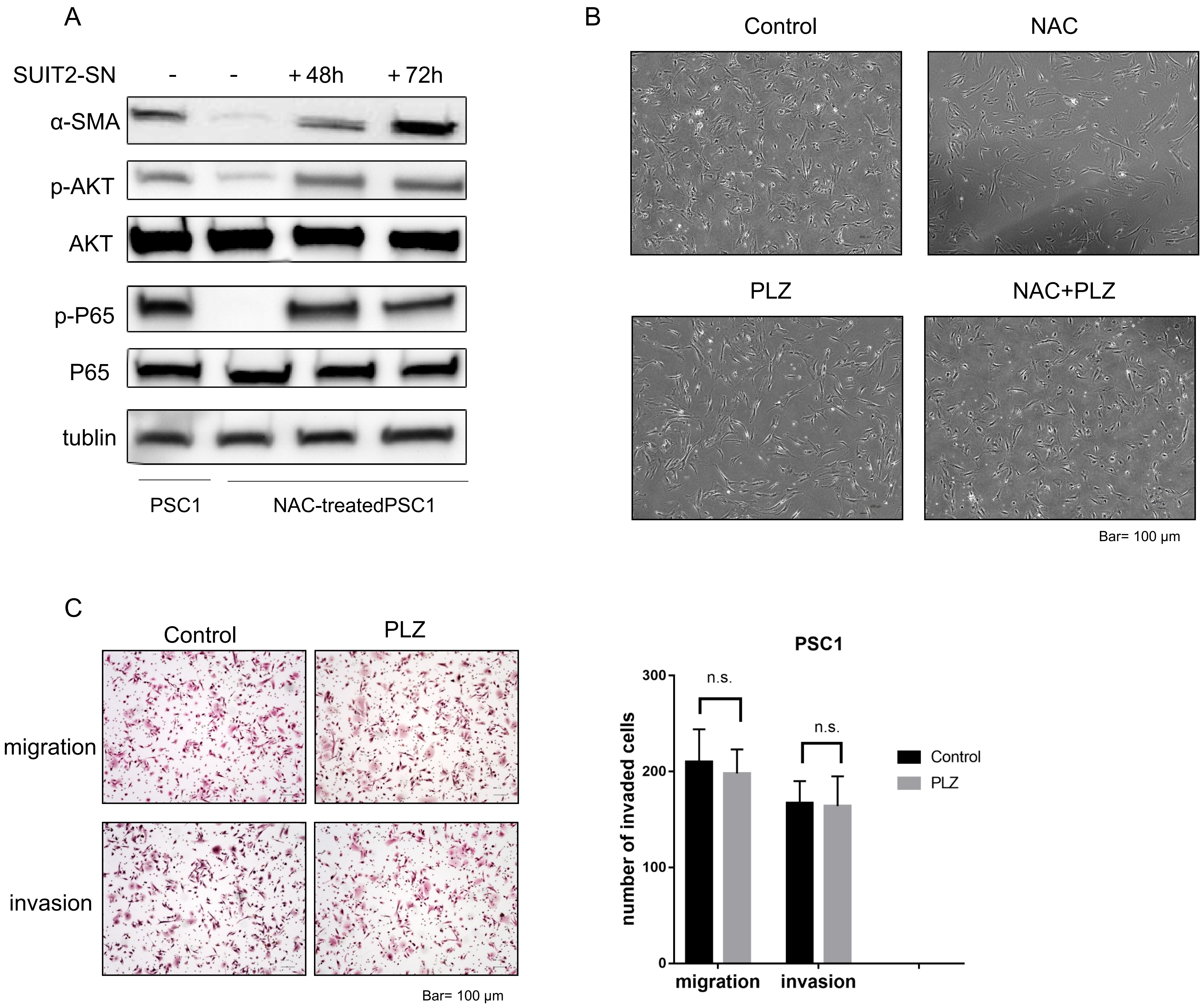

A
SUIT2-SN
-
-
+ 48h
+ 72h
α-SMA
p-AKT
AKT
p-P65
P65
tublin
PSC1
NAC-treatedPSC1
B
Control
NAC
PLZ
NAC+PLZ
Bar= 100 μm
C
Control
PLZ
migration
invasion
Bar= 100 μm
PSC1
number of invaded cells
300
200
100
0
n.s.
n.s.
Control
PLZ
migration
invasion

D

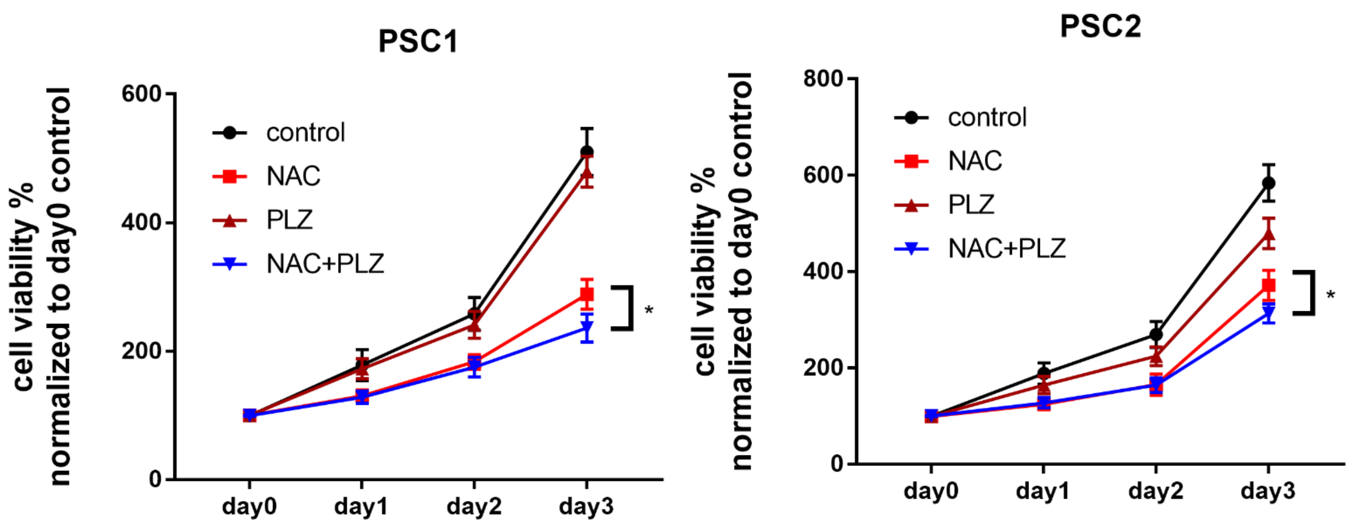
PSC1
cell viability %
normalized to day0 control
600
400
200
0
day0
day1
day2
day3
control
NAC
PLZ
NAC+PLZ
*
PSC2
cell viability %
normalized to day0 control
800
600
400
200
0
day0
day1
day2
day3
control
NAC
PLZ
NAC+PLZ
*

E

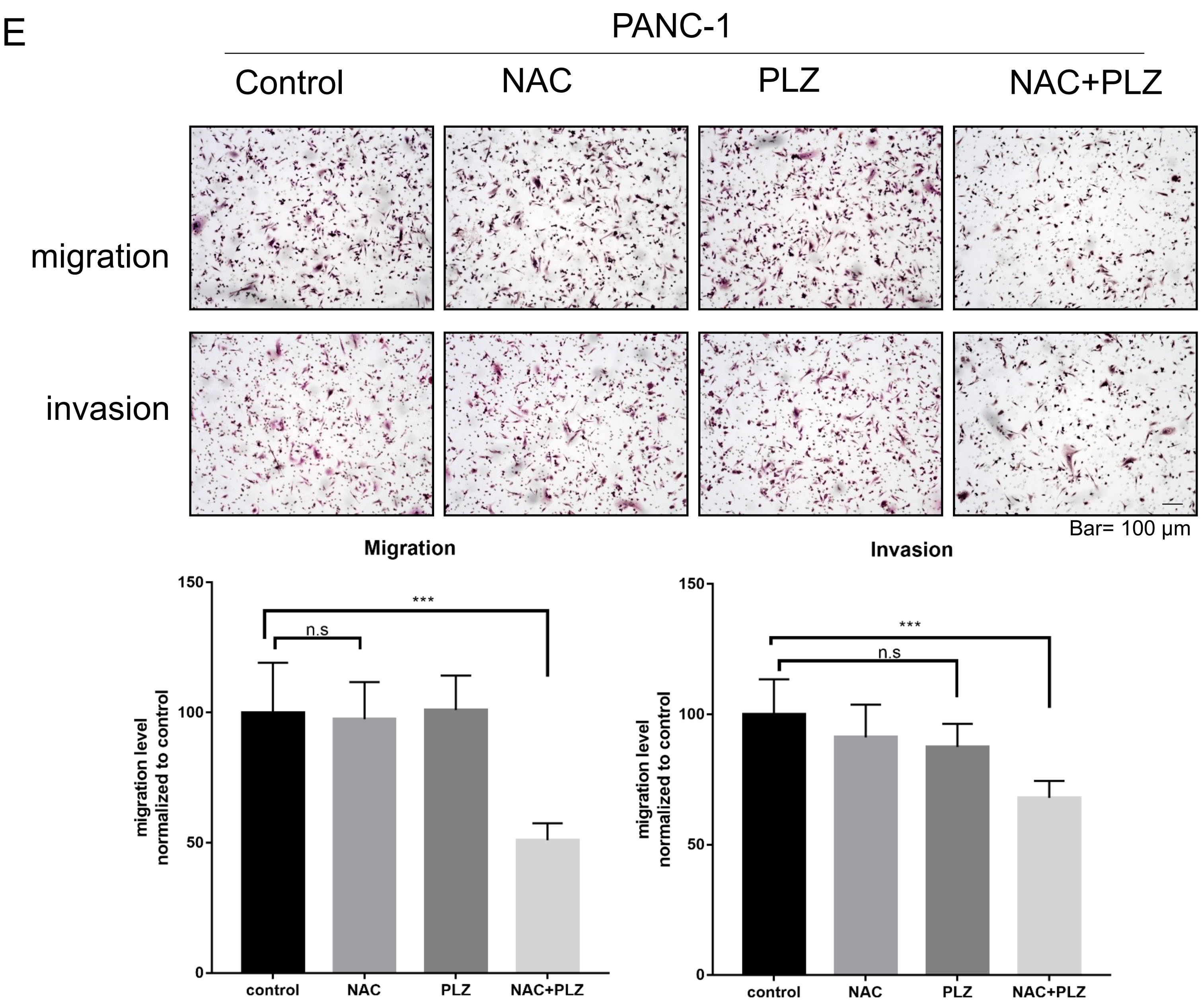
PANC-1
Control
NAC
PLZ
NAC+PLZ
migration
invasion
Bar= 100 μm
Migration
migration level
normalized to control
150
100
50
0
control
NAC
PLZ
NAC+PLZ
n.s
***
Invasion
migration level
normalized to control
150
100
50
0
control
NAC
PLZ
NAC+PLZ
n.s
***

Figure S8

A

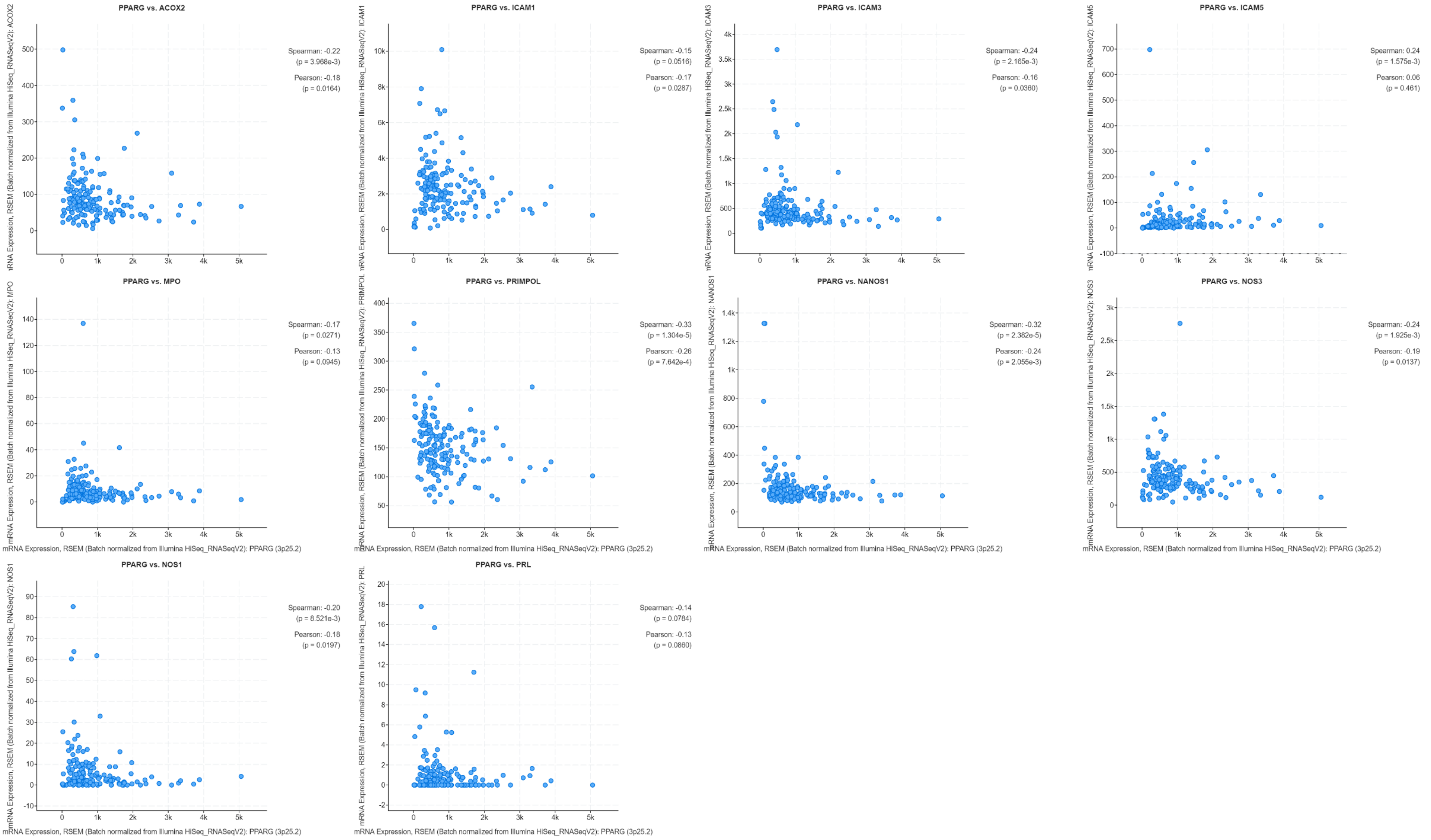
PPARG vs. ACOX2
Spearman: -0.22 (p = 3.968e-3)
Pearson: -0.18 (p = 0.0164)
PPARG vs. ICAM1
Spearman: -0.15 (p = 0.0516)
Pearson: -0.17 (p = 0.0287)
PPARG vs. ICAM3
Spearman: -0.24 (p = 2.165e-3)
Pearson: -0.16 (p = 0.0360)
PPARG vs. ICAM5
Spearman: 0.24 (p = 1.575e-3)
Pearson: 0.06 (p = 0.461)
PPARG vs. MPO
Spearman: -0.17 (p = 0.0271)
Pearson: -0.13 (p = 0.0945)
PPARG vs. PRIMPOL
Spearman: -0.33 (p = 1.304e-5)
Pearson: -0.26 (p = 7.642e-4)
PPARG vs. NANOS1
Spearman: -0.32 (p = 2.382e-5)
Pearson: -0.24 (p = 2.055e-3)
PPARG vs. NOS3
Spearman: -0.24 (p = 1.925e-3)
Pearson: -0.19 (p = 0.0137)
PPARG vs. NOS1
Spearman: -0.20 (p = 8.521e-3)
Pearson: -0.18 (p = 0.0197)
PPARG vs. PRL
Spearman: -0.14 (p = 0.0784)
Pearson: -0.13 (p = 0.0860)
mRNA Expression, RSEM (Batch normalized from Illumina HiSeq_RNASeqV2): PPARG (3p25.2)

B

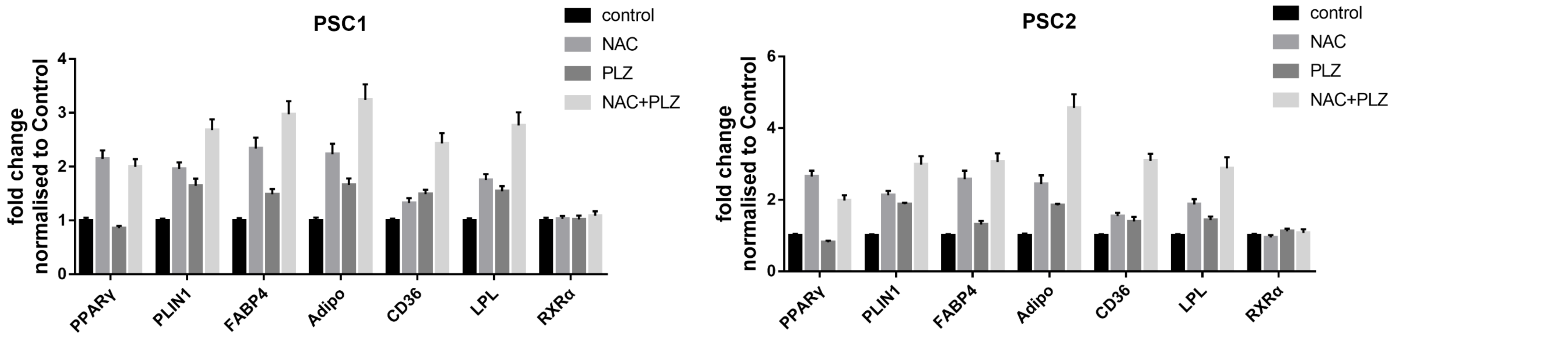
PSC1
PSC2
control
NAC
PLZ
NAC+PLZ
fold change normalised to Control
PPARγ
PLIN1
FABP4
Adipo
CD36
LPL
RXRα

Figure S9

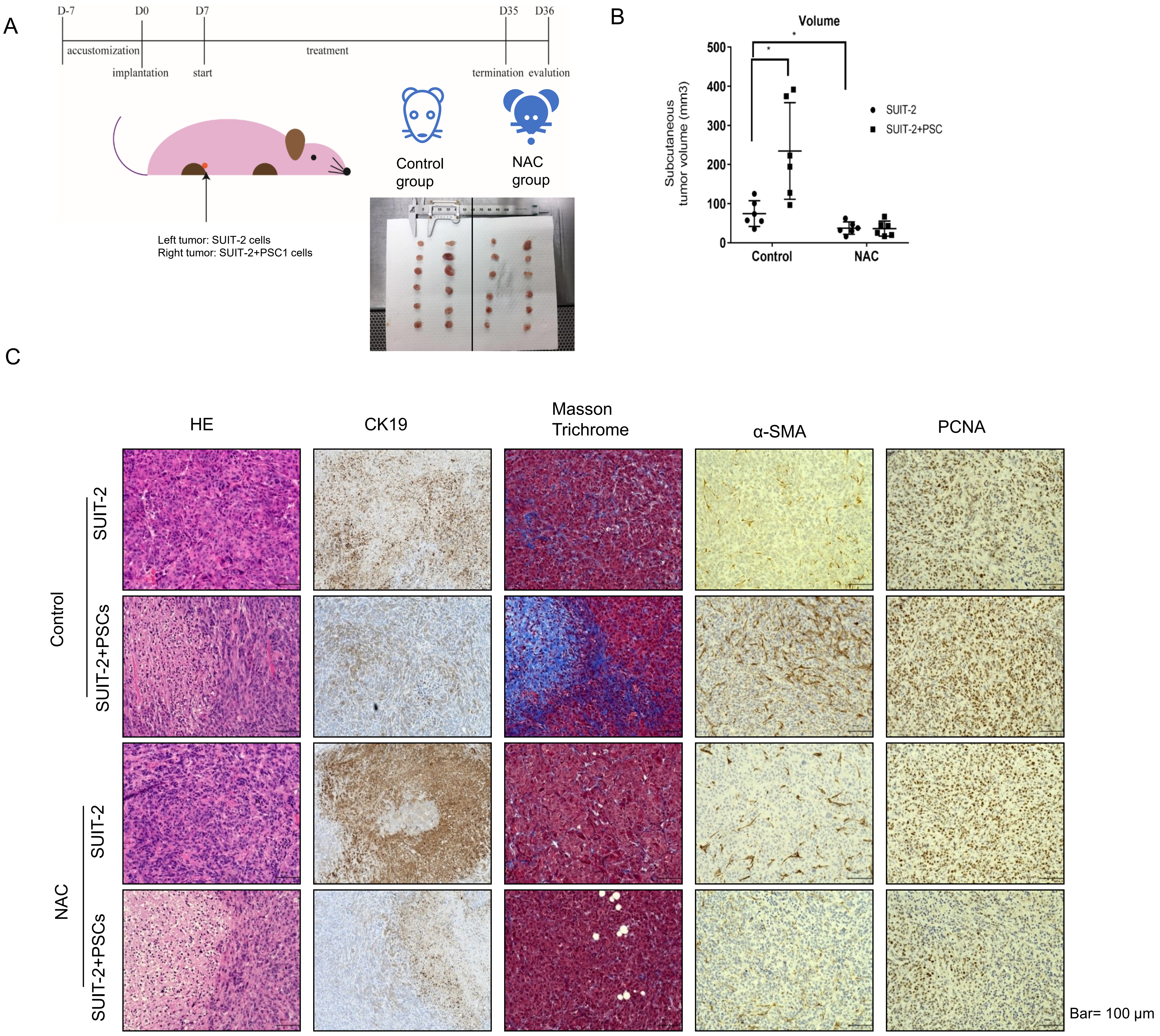
A
D-7
D0
D7
D35
D36
accustomization
treatment
implantation
start
termination
evalution
Control group
NAC group
Left tumor: SUIT-2 cells
Right tumor: SUIT-2+PSC1 cells
B
Volume
Subcutaneous tumor volume (mm3)
500
400
300
200
100
0
*
*
SUIT-2
SUIT-2+PSC
Control
NAC
C
HE
CK19
Masson Trichrome
α-SMA
PCNA
Control
SUIT-2
SUIT-2+PSCs
NAC
SUIT-2
SUIT-2+PSCs
Bar= 100 μm

D

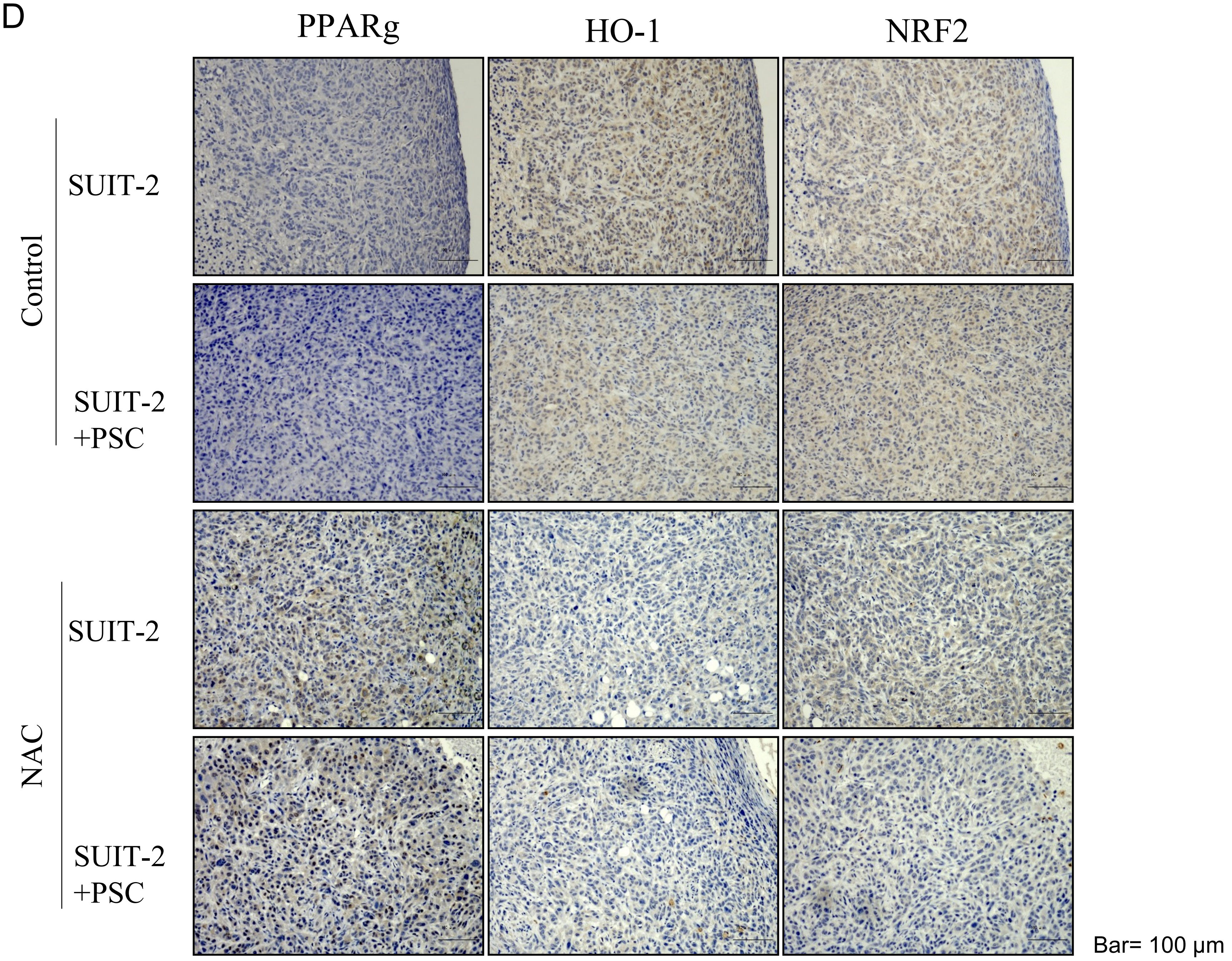

PPARg
HO-1
NRF2
Control
SUIT-2
SUIT-2
+PSC
NAC
SUIT-2
SUIT-2
+PSC
Bar= 100 μm

E

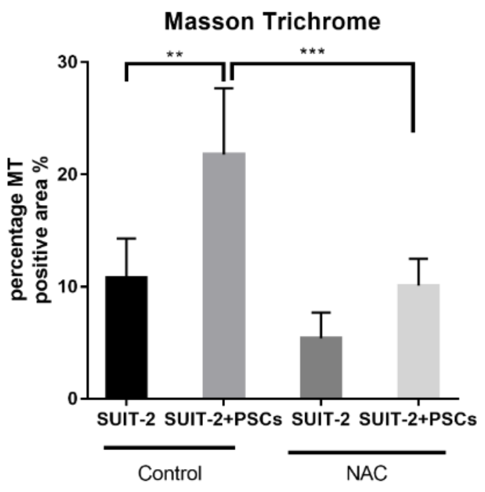

Masson Trichrome
**
***
percentage MT positive area %
30
20
10
0
SUIT-2
SUIT-2+PSCs
SUIT-2
SUIT-2+PSCs
Control
NAC

F

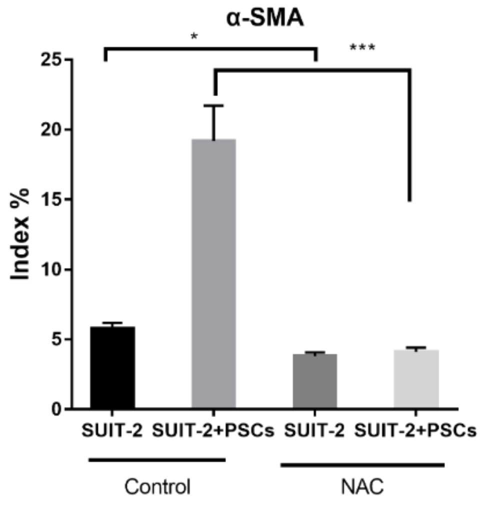

α-SMA
*
***
Index %
25
20
15
10
5
0
SUIT-2
SUIT-2+PSCs
SUIT-2
SUIT-2+PSCs
Control
NAC

G

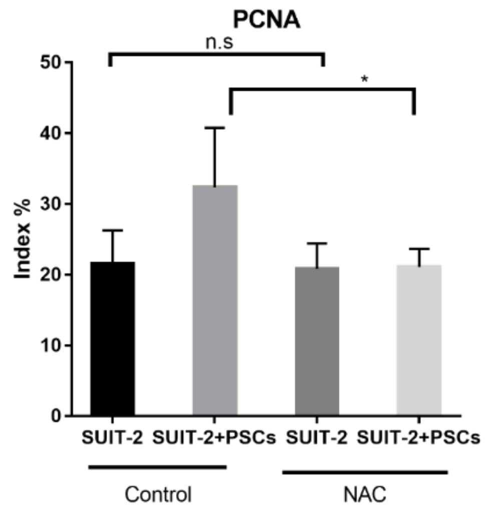

PCNA
n.s
*
Index %
50
40
30
20
10
0
SUIT-2
SUIT-2+PSCs
SUIT-2
SUIT-2+PSCs
Control
NAC

Figure S10

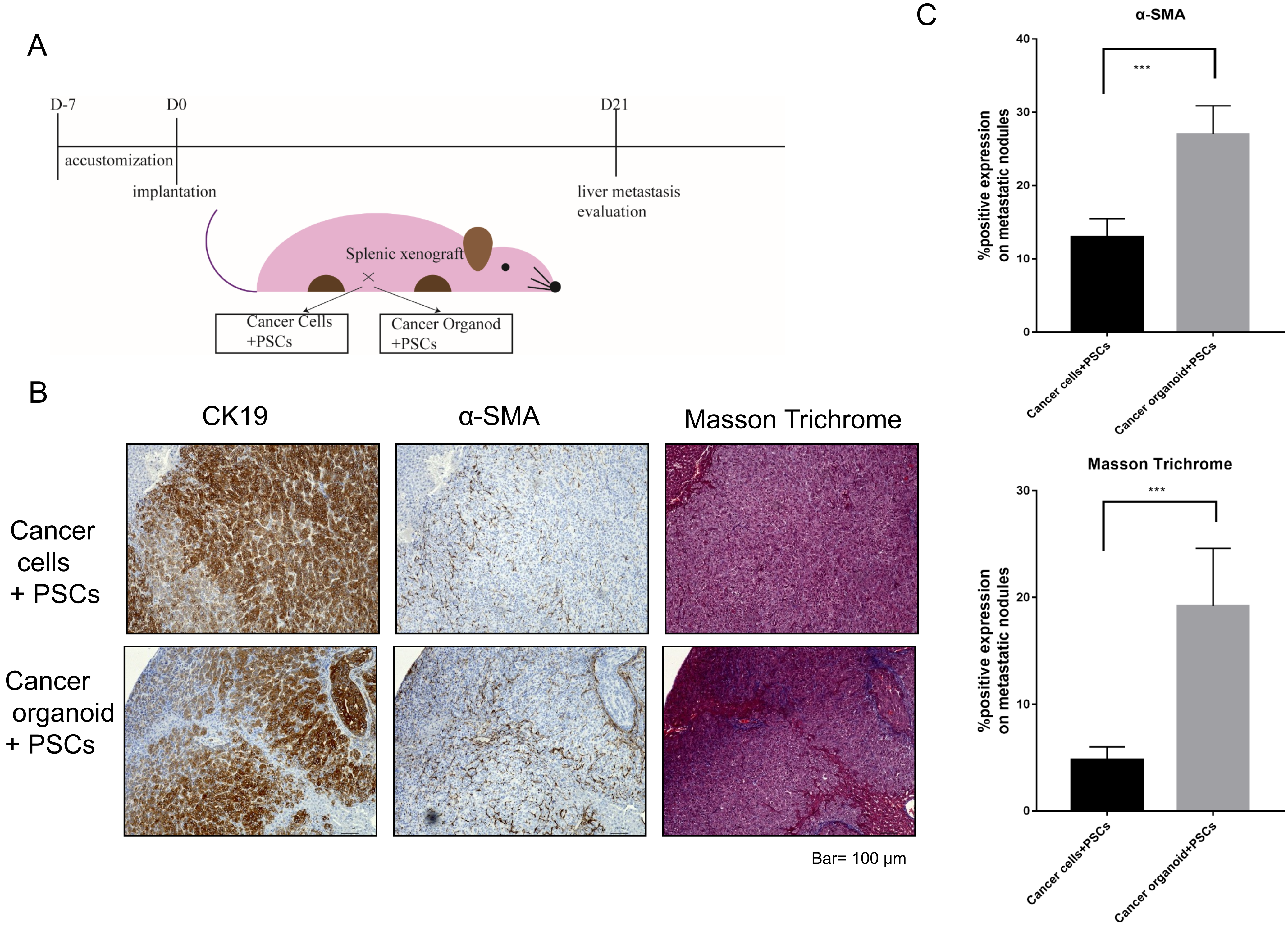
A
D-7
D0
D21
accustomization
implantation
liver metastasis evaluation
Splenic xenograft
Cancer Cells +PSCs
Cancer Organod +PSCs
B
CK19
α-SMA
Masson Trichrome
Cancer cells + PSCs
Cancer organoid + PSCs
Bar= 100 μm
C
α-SMA
%positive expression on metastatic nodules
0
10
20
30
40
***
Cancer cells+PSCs
Cancer organoid+PSCs
Masson Trichrome
%positive expression on metastatic nodules
0
10
20
30
***
Cancer cells+PSCs
Cancer organoid+PSCs

Figure S11

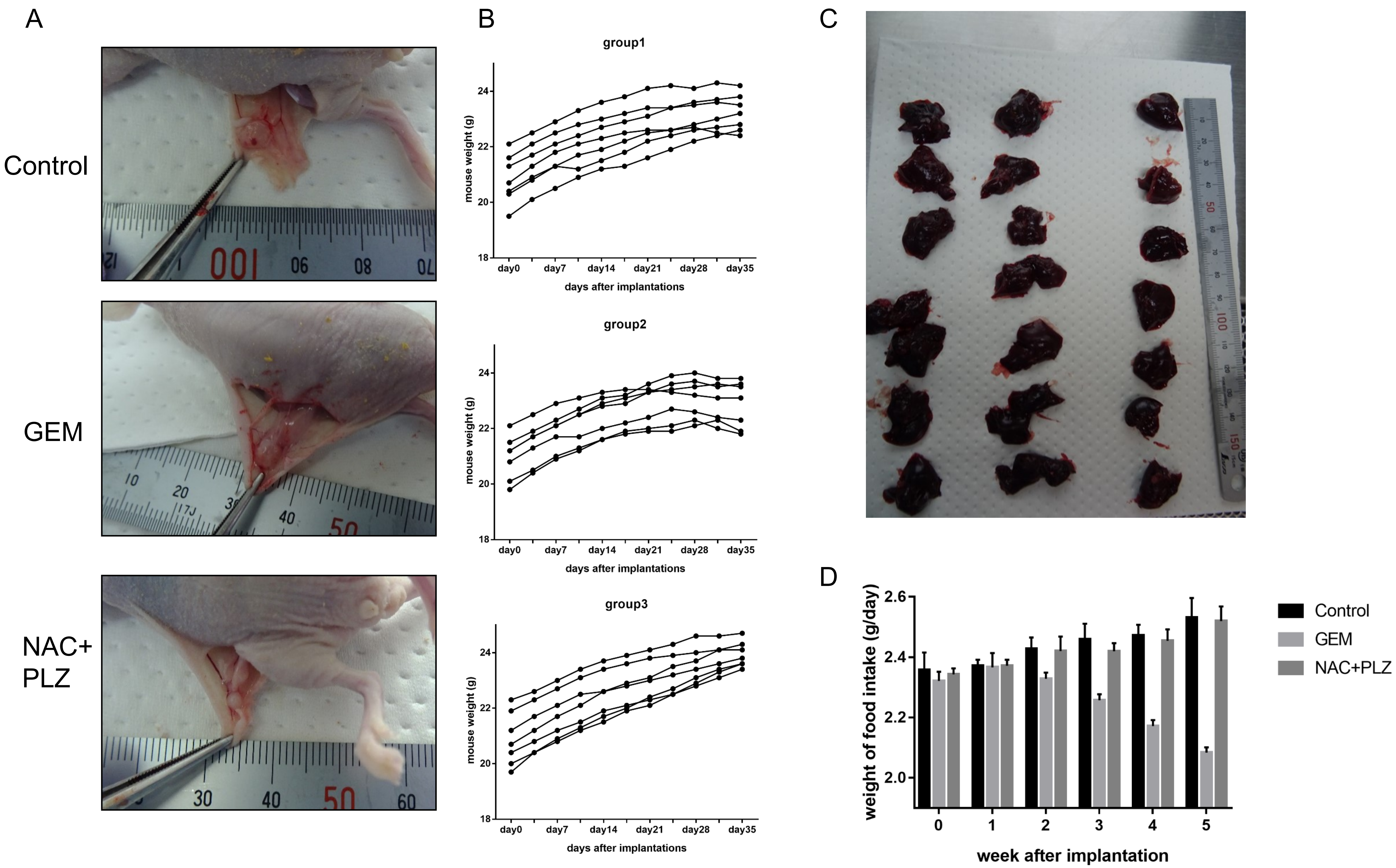
A
Control
GEM
NAC+
PLZ
B
group1
group2
group3
mouse weight (g)
18
20
22
24
day0
day7
day14
day21
day28
day35
days after implantations
C
D
weight of food intake (g/day)
2.0
2.2
2.4
2.6
0
1
2
3
4
5
week after implantation
Control
GEM
NAC+PLZ

E

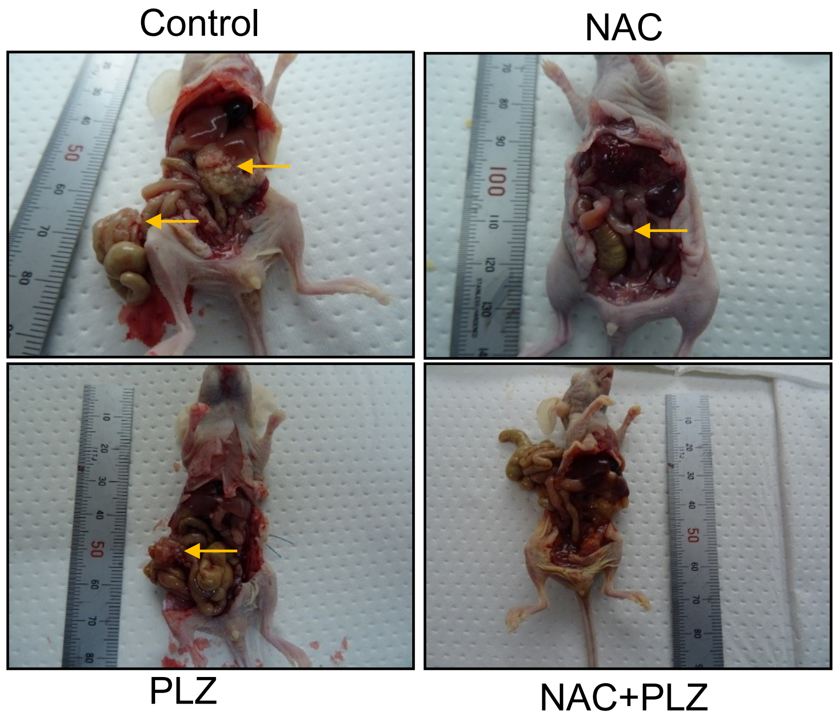
Control
NAC
PLZ
NAC+PLZ

F

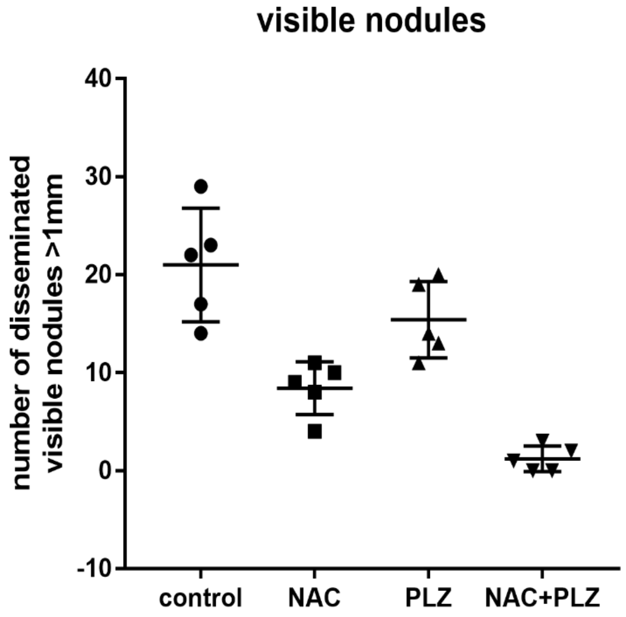
visible nodules
number of disseminated visible nodules >1mm
40
30
20
10
0
-10
control
NAC
PLZ
NAC+PLZ

G

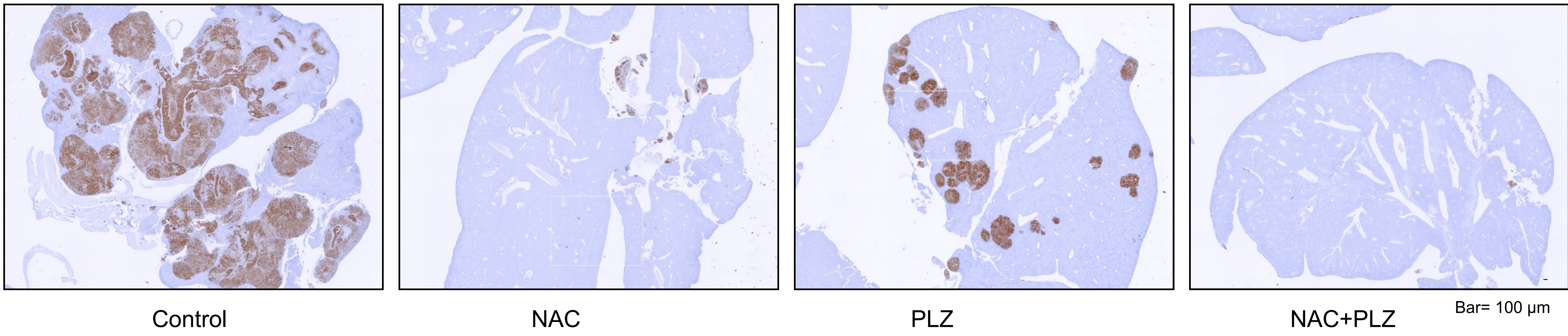
Control
NAC
PLZ
NAC+PLZ
Bar= 100 μm
